# oFVSD: a Python package of optimized forward variable selection decoder for high-dimensional neuroimaging data

**DOI:** 10.3389/fninf.2023.1266713

**Published:** 2023-09-26

**Authors:** Tung Dang, Alan S. R. Fermin, Maro G. Machizawa

**Affiliations:** ^1^Center for Brain, Mind, and KANSEI Sciences Research, Hiroshima University, Hiroshima, Japan; ^2^Graduate School of Agricultural and Life Sciences, The University of Tokyo, Tokyo, Japan

**Keywords:** machine learning, forward variable selection, optimized hyperparameter, neural decoding, MRI, VBM (voxel-based morphometry)

## Abstract

The complexity and high dimensionality of neuroimaging data pose problems for decoding information with machine learning (ML) models because the number of features is often much larger than the number of observations. Feature selection is one of the crucial steps for determining meaningful target features in decoding; however, optimizing the feature selection from such high-dimensional neuroimaging data has been challenging using conventional ML models. Here, we introduce an efficient and high-performance decoding package incorporating a forward variable selection (FVS) algorithm and hyper-parameter optimization that automatically identifies the best feature pairs for both classification and regression models, where a total of 18 ML models are implemented by default. First, the FVS algorithm evaluates the goodness-of-fit across different models using the k-fold cross-validation step that identifies the best subset of features based on a predefined criterion for each model. Next, the hyperparameters of each ML model are optimized at each forward iteration. Final outputs highlight an optimized number of selected features (brain regions of interest) for each model with its accuracy. Furthermore, the toolbox can be executed in a parallel environment for efficient computation on a typical personal computer. With the optimized forward variable selection decoder (oFVSD) pipeline, we verified the effectiveness of decoding sex classification and age range regression on 1,113 structural magnetic resonance imaging (MRI) datasets. Compared to ML models without the FVS algorithm and with the Boruta algorithm as a variable selection counterpart, we demonstrate that the oFVSD significantly outperformed across all of the ML models over the counterpart models without FVS (approximately 0.20 increase in correlation coefficient, *r*, with regression models and 8% increase in classification models on average) and with Boruta variable selection algorithm (approximately 0.07 improvement in regression and 4% in classification models). Furthermore, we confirmed the use of parallel computation considerably reduced the computational burden for the high-dimensional MRI data. Altogether, the oFVSD toolbox efficiently and effectively improves the performance of both classification and regression ML models, providing a use case example on MRI datasets. With its flexibility, oFVSD has the potential for many other modalities in neuroimaging. This open-source and freely available Python package makes it a valuable toolbox for research communities seeking improved decoding accuracy.

## 1. Introduction

Neuroimaging data such as structural and functional magnetic resonance imaging (MRI) data provide information about the functional neuroanatomy with a high spatial resolution and play an essential role in providing researchers with unprecedented access to the inner workings of the brain of a healthy individual or an individual with a neurological disease or psychiatric disorder (Zhu et al., 2019). Identifying brain regions that differentiate healthy and nonhealthy participants (Nielsen et al., 2020) or the prognosis of patients’ pathological states (Janssen et al., 2018) is essential for neuroscientific studies. Needless to say, the impact of those findings is rooted in the accuracy of their decoding.

In the last couple of decades, we have seen a surge of studies turning to ML models to extract exciting new information from neuroimaging data. For example, the partial least squares (PLS) model was proposed to extract distributed neural signal changes by taking advantage of image elements’ spatial and temporal dependencies (McIntosh et al., 1996; McIntosh and Lobaugh, 2004). The adaptive boosting model (‘AdaBoost’) was proposed to classify addiction disorder patients and healthy controls based on observed 3-dimensional functional brain images (Warren and Moustafa, 2022). The existence of diverse ML models has raised the question of which and when an ML model is better suited to extract important new information from neuroimaging data (O’Toole et al., 2007; Pereira et al., 2009). However, the selection of the most appropriate models for a specific dataset and purposes is challenging for people with little experience in ML since the appropriate choice of a model depends on the number of features (Saeys et al., 2007).

The curse of dimensionality of neuroimaging data can negatively affect the generalization performance of ML models, leading to estimation instability, model overfitting, local convergence, and large estimation errors (Mwangi et al., 2014). For example, the decoding abilities of models that depend on specific distributions of data, such as geometric distributions of data, can be significantly influenced in the high-dimensional data space. A naïve learning model requires the number of training data points to be an exponential function of the attribute dimension (Jain et al., 2000). Furthermore, the problem of the high dimensionality of neuroimaging data (e.g., an extremely large number of voxels in fMRI research) poses a number of challenges (Vul et al., 2009) even if a model is based on nonparametric strategies (Huang et al., 2012). For example, in the random forest model, available features are randomly sampled to generate different subspaces of features used to train each decision tree in an ensemble (Kuncheva et al., 2010). Because it is typical to observe only a few features out of many that significantly contribute to the performance of these models, a large number of irrelevant features appear in these subspaces. Thus, the average strength of decision trees can be diluted, thereby increasing the generalization error of the random forest model. These problems also exist in neural network models when the high dimensionality of data confounds learning techniques, and the network must allocate its resources to represent many irrelevant components (Scott, 1992).

Recent advancements in neuroimaging technologies have also increased the data size; namely, the total number of features to be considered has increased. Therefore, a feature reduction technique has become one of the essential aspects of neuroimaging research (Mwangi et al., 2014). The size of neuroimaging data may lead to a computational burden. However, building a pipeline for hundreds of thousands of brain regions can be very costly and time-consuming. Recently, several ML methods incorporating parallel computing environments have been developed (Xing et al., 2016); implementing a fast and efficient pipeline would be of potential application for analyzing a large amount of neuroimaging data.

While many algorithms have been proposed, one thorough ML model is forward variable selection (FVS). The FVS algorithm is a member of the stepwise feature selection algorithm family (Guyon and Elisseeff, 2003; Kutner et al., 2005; Weisberg, 2005; Chandrashekar and Sahin, 2014). It is also one of the first and most popular algorithms for causal feature selection in some fields, such as gene selection, microarray data analysis, and gene expression data analysis (Ooi and Tan, 2003; Blanco et al., 2004; Jirapech-Umpai and Aitken, 2005; Saeys et al., 2007). The powerful nature of feature decoding in the analysis of high-dimensional microbiome data has also been demonstrated (Dang et al., 2022; Dang and Kishino, 2022). The FVS can be a powerful additional tool for neuroimaging research.

Despite a significant rise in the application of ML models and their potential contributions to understanding brain functions, neuroimaging data are ill-posed to the high-dimensionality problem. Here, we propose a state-of-the-art and effective ML package as a solution to the high-dimensionality problem of neuroimaging data that is easy to use by neuroscientists interested in applying ML models to decode their neuroimaging data with little computational programming. In this study, we developed a novel decoding pipeline to overcome these challenges by combining two frameworks. First, we developed an ML framework incorporating an FVS algorithm that integrates model selection steps to detect the minimal set of features that could maximize the predictive performance. Second, the pipeline selects the best model from a predetermined set of regression and classifier models. This simple yet comprehensive two-stage algorithm automatically and effectively identifies important features from neuroimaging data. Moreover, because the nature of the FVS is computationally intensive and time-costly, the toolbox executes in a parallel environment to save computational costs. As a proof of concept of our approach to neuroimaging datasets, structural neuroimaging data were acquired to examine the feasibility of our proposed FVS toolbox to decode the neuroanatomical representation of (1) biological sex and (2) age with binary classification and multiple regression models, respectively.

## 2. Methods and material

### 2.1. Forward variable selection (FVS) algorithm

The FVS algorithm requires an ML model for feature selection and uses its performance to evaluate and determine which features are selected. The key idea behind the FVS algorithm is to select a feature that provides the largest improvement in terms of the predictive performance of the ML model and append this feature to the set of selected features in each forward iteration. The iterations stop when there is no feature improvement in the performance upon adding a feature or the maximum number of selected features has been reached.

**Figure 1:**
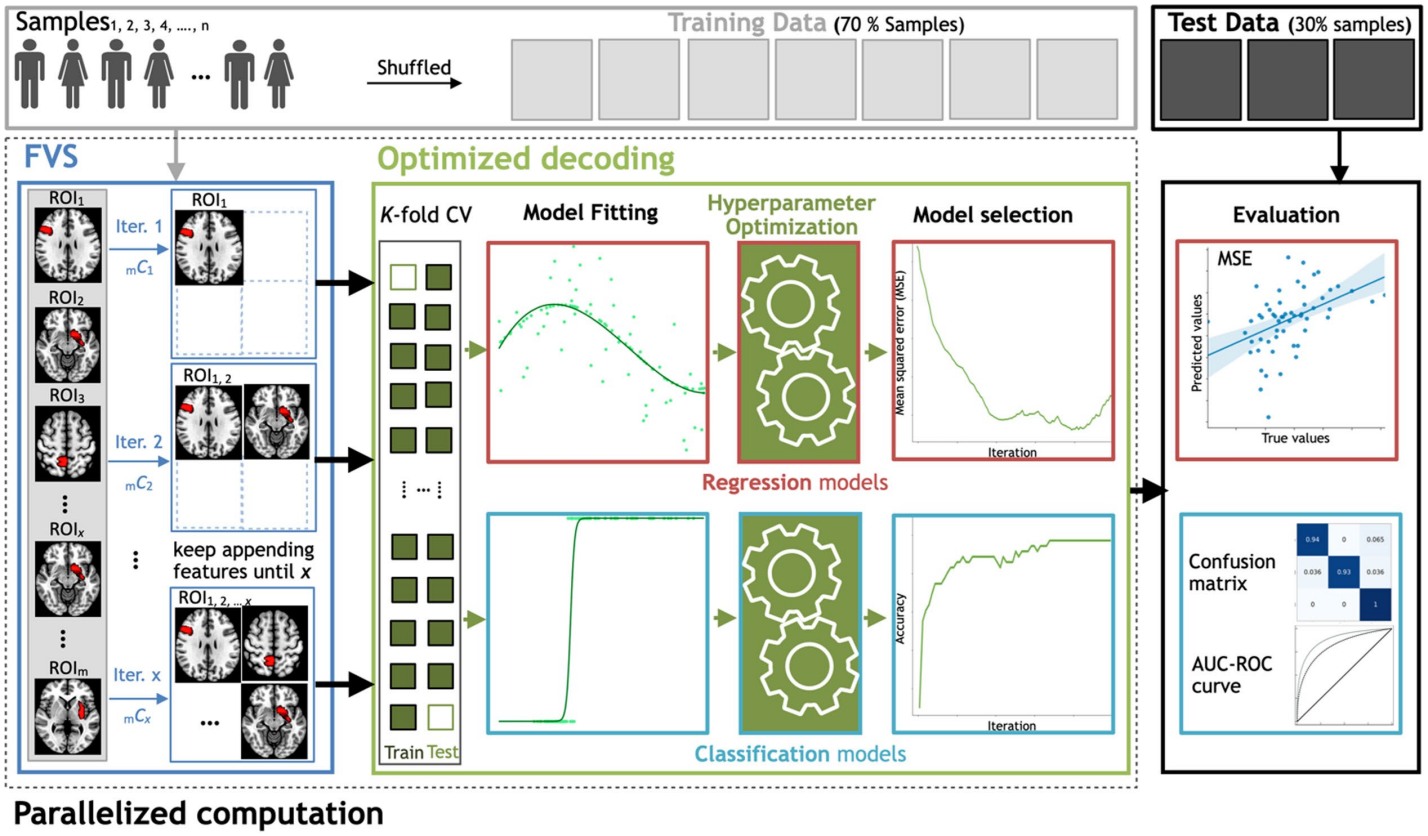
Workflow schematics of the automatic ML toolbox coupled with the forward variable selection (FVS) algorithm. All features, i.e., the gray matter volume data from each region of interest (ROI), undergo the FVS step, followed by either regression-based or classification-based ML with K-fold cross-validation (CV). The random search and grid search strategies with cross-validation were adopted to optimize the hyperparameters of the ML models at each iteration of the FVS algorithm. The final outcomes were evaluated based on the MSE and MAE for regression-based models and the AUC and confusion matrix for classification-based model. *n* is the number of samples, m is the total number of ROIs (246 ROIs in this study) and x is the number of ROIs that the user wants to select.

In this proof of concept to decode either sex or age from regional gray matter volume, we used the FVS algorithm to identify a small number of features (i.e., regions of interest) to improve the performance of ML models in the subsequent step of regression or classification. At each learning step, a brain region that provides the largest increase in the predictive performance of regression or classification models is selected and added to the set of selected brain regions. This process continues until there is no further performance improvement after selecting a brain region or the maximum number of selected brain regions that have been set *a priori* has been reached. To save computational time or to restrict the number of to-be-selected features for a certain purpose, users should specify a maximum number of brain regions for the FVS algorithm. If not, a maximum value is all the available brain regions in the data (246 ROIs). In this study, 100 ROIs is the maximum number of brain regions for the FVS algorithm. Model selection for brain region signature identification can also be performed using the FVS algorithm (see Figure 1). At each forward iteration, given the selected brain regions, samples were randomly split into a training dataset comprising 70% of the samples and a test dataset comprising the remaining 30% of the samples. To select an appropriate model configuration for a specific task, such as a prediction of age, fivefold cross-validation was performed on the training data to optimize the respective hyperparameters. Two standard algorithms of hyperparameter optimization, the grid search and random search strategies with cross-validation, were implemented to select the best values for the parameters of the ML model (Bisong, 2019; Agrawal, 2021). The best-performing hyperparameters for each model were achieved when the MSE was minimized.

Based on the specific numbers of parameters of the ML model, grid search with cross-validation, all parameter combinations are exhaustively considered, while with the random search with cross-validation, a given number of values are randomly selected from a parameter space was considered for parameter optimization (Bergstra and Bengio, 2012). These search strategies suffer from high-dimensional spaces but can often easily be parallelized since the hyperparameter values that the algorithm works with are usually independent of each other. Therefore, the ML models have a large number of parameters, such as the random forest and decision tree models. The random search with a cross-validation strategy is used to balance computational time and predictive accuracy. The grid search with cross-validation strategy is used for ML models with a few parameters, such as the lasso or ridge models. Following the hyperparameter optimization, the best ML model is specified, as well as the final selected features, namely brain regions in this case.

The FVS algorithm is implemented in a parallel computing environment to reduce the computational burden in terms of time cost. A number of packages provide high-performance computing solutions in Python (Palach, 2014). We used the thread-based parallelism and process-based parallelism that is provided in the joblib package to separate Python worker processes to execute tasks on separate CPUs. To parallelize each FVS iteration, the input variables were separated randomly into a number of subsets. Because of the high dimensionality of neuroimaging data, the number of these subsets (or comparisons) is usually larger than the number of processors in a single computer system. In a previous study, a computer-friendly procedure was proposed for very high-dimensional microbiome data (Dang and Kishino, 2022). To introduce efficient computation, queues were created to randomly assign subsets to each processor that runs the computational processes from its own privately prepared queue (Dang and Kishino, 2022).

As the counterpart feature selection method we propose, the Boruta algorithm (Kursa and Rudnicki, 2010) was tested to compare the performance of the FVS. Kursa and Rudnicki (2010) originally developed the wrapper algorithm to identify all important variables within a classification framework. The Boruta feature selection algorithm is applied in bioinformatics areas to select protein targets (Pietzner et al., 2021; Al-Nesf et al., 2022), microbial functions (Diamond et al., 2019; Saffouri et al., 2019; Edwinson et al., 2022), and metabolomic profiles (Metwaly et al., 2020; Mayneris-Perxachs et al., 2022). The main idea of the Boruta algorithm is to create shadow features by randomly permuting the values of each original feature. This permutation is to generate a null distribution that represents the expected importance scores of features. Then, the original features and their corresponding shadow features are used to train the random forest classifier. The importance of each original and shadow feature is determined based on the random forest model. The z-score of the original feature is then computed by comparing its importance score with the distribution of importance scores of the corresponding shadow features. If the z-score is significantly higher than the expected chance level, it indicates that the original feature is more important than the shadow features. In this study, the maximum value is all the available brain regions in the data (246 ROIs) for the Boruta algorithm.

### 2.2. Regression-based ML models

We explored various ML models to examine how a regional brain structure could contain information representing age. After the preprocessing of structural imaging data, the input (target features) included high-dimensional structural gray matter volumetric data from 1,113 samples in 246 brain regions (see Section 3 for details). We applied a variety of ML models to identify features and to reduce the dimension of this input with parametric regularization models for feature selection and nonparametric models that perform a random sampling of the available features to generate different subspaces of features to achieve a trade-off between bias and variance. In our toolbox, we selected two nonparametric models and 10 parametric models.

For the nonparametric regression models, the commonly used decision tree regression, random forest (RF), and Gaussian process models were selected (Hastie et al., 2009). (1) Decision tree regression is a supervised learning model that sets up a decision rule depending on the features at every interior node (Hastie et al., 2009). The features selected for the first partition at the root have the largest relevance. This feature selection procedure is recursively repeated for each subset at the node until further partitioning becomes impossible. The decision tree regression is typically considered to analyze MRI images (Naik and Patel, 2014; Filli et al., 2018; Kim et al., 2018). (2) The random forest (RF) is a modification of the bagging regression that aggregates a large collection of decision trees (Breiman, 2001). The primary step in building an ensemble of decision trees is to randomly sample the available features to generate different subspaces of features at each node of each unpruned decision tree. Using this strategy, better estimation performances can be obtained compared with using a single decision tree because each tree estimator has a low bias but high variance, whereas the bagging process of RF achieves a bias-variance trade-off. The random forest (RF) model has become a standard data analysis tool in multiple areas, such as bioinformatics (Boulesteix et al., 2012; Ferreira and Figueiredo, 2012) and neuroimaging analysis (Mitra et al., 2014; Eshaghi et al., 2016). (3) The Gaussian process (GP) is a nonparametric model that is a natural generalization of a multivariate Gaussian distribution to a Gaussian distribution over a specific family of functions, such as kernel functions (Rasmussen, 2003). In GP regression, a prior distribution is proposed directly over the nonlinear function space rather than specifying a parametric family of nonlinear functions. Different kernels can be used to express different structures observed in the data. Thus, the GP has a large degree of flexibility in capturing the underlying signals without imposing strong modeling assumptions. This property makes the GP an attractive model for analyzing genetic data (Chu et al., 2005) as well as MRI data (Wassermann et al., 2010).

We selected eight parametric ML models, including ‘ridge regression,’ ‘least absolute shrinkage and selection operator (Lasso) regression,’ ‘kernel ridge regression,’ ‘multitask Lasso regression, least angle regression (Lar),’ ‘LassoLar regression,’ ‘elastic net regression,’ and ‘regularized linear model with stochastic gradient descent (SGD).’ (1) Ridge regression is a linear least squares model that uses L2 regularization or weight decay to control the relative importance of features (Hastie et al., 2009). L2 regularization encourages weight values to decay toward zero. Thus, ridge regression can be used to overcome the disadvantages of the ordinary least square method, i.e., the variance in the estimate of the linear transform may be large because the number of features is significantly larger than the number of samples. (2) Least absolute shrinkage and selection operator (Lasso) regression, which is another type of linear regression, uses L1 regularization and can eliminate a number of coefficients from the model by adding a penalty equal to the absolute value of their magnitude (Hastie et al., 2009). (3) Kernel ridge regression is an extension of ridge regression that is used when the number of dimensions can be much larger, or even infinitely larger, than the number of samples (Vovk, 2013). The main idea is to propose the kernel trick to convert the original data space into the fancy feature space that can significantly reduce the computational burden of learning processes. (4) Multitask Lasso regression generalizes the Lasso to the multitask setting by replacing the L1-norm regularization term with the sup-norm regularization sum (Hastie et al., 2009). (5) The least angle regression (Lar) model is the modification of the Lasso and the forward stagewise linear regression models, where the number of features is significantly greater than the number of samples (Efron et al., 2004). At each iteration, Lars selects the feature most correlated with the target. If multiple features have a similar correlation, the direction equiangular between the features is moved forward. (6) The Lasso model fit with least angle regression (LassoLar) is the combination of Lar and Lasso and is implemented to improve the variable selection (Efron et al., 2004). (7) The elastic net model is the generalization of ridge regression and lasso. This model proposes the elastic net penalty, which controls the coefficients’ balance between the L1 and L2 regularization (Zou and Hastie, 2005; Hastie et al., 2009). Thus, an elastic net can be used to perform feature selection in a high-dimensional space. And (8) Regularized linear model with stochastic gradient descent (SGD) learning is an extension of the ridge, Lasso, and elastic net models with a large number of training samples implemented with a plain stochastic gradient descent learning routine (Hastie et al., 2009). See the Supplementary material for detailed explanations for each ML model.

For each ML model, the parameter optimization steps were developed. Specifically, in the cases of the ridge, Lasso, multitask Lasso, Lar, and LassoLar models, the complexity of the parameters that control the amount of shrinkage was optimized. The elastic net model includes an additional parameter that controls a combination of L1 and L2 penalties separately. The main parameters of the regularized linear model with SGD learning were loss functions, penalty options, and the learning rate schedule, whereas those of the kernel ridge regression model were kernel options that include linear, Laplacian, Gaussian, and sigmoid kernels, the regularization parameter, and the kernel coefficient, and those of the decision tree model were the maximum depth of the tree, the minimum number of samples required to split an internal node, the minimum number of the samples required to be at a leaf node, the function to measure the quality of a split, the strategy used to choose the split at each node, and the number of features to consider when looking for the best split. The parameters of the random forest regression were the same as those of the decision tree regression, except that the number of trees was an additional parameter.

As a criterion to compare the performance of regression, the mean squared error (MSE), mean absolute error (MAE), and Spearman correlation coefficients that were calculated between the predicted and the true values were calculated (Hastie et al., 2009). In the field of statistics, the Akaike or Bayesian information criteria (also known as AIC or BIC, respectively) are widely used indices to quantify the fit of a model (Burnham and Anderson, 2004); however, these information criteria methods do not apply for nonparametric regression models (e.g., decision tree, random forest). Thus, we selected the MSE and MAE, which are applicable across all models.

### 2.3. Classification-based ML models

We also extended the application of our model to study the binary or multi-class classification. We examined how brain structure could contain information representing sex (male or female). The automated classification models include nonparametric models, such as decision tree, random forest, gradient boosting models, extreme gradient boosting, and extremely randomized trees, which have the capability of regression and classification. (1) Decision trees are commonly utilized classification models in various fields, such as machine learning and data mining (Gavankar and Sawarkar, 2017). Decision trees include a number of tests or attribute nodes linked to subtrees and decision nodes labeled with a class, i.e., a decision. A sample is classified by starting at the root node of the tree. Each node represents features in a group to be classified, and each subset defines a value that can be taken by the node (Hastie et al., 2009). The entropy, Gini index, and information gain are the standard measures of a dataset’s impurity or randomness in decision tree classification. (2) Random forest classification is one of the most popular ensemble models that can be used to avoid the tendency of simple decision trees to overfit (Breiman, 2001). Similar to regression, random forest classification proposes a slightly randomized training process to build multiple decision trees independently. The randomization processes include using only a random subset of the whole training dataset to build each tree and using a random subset of the features or a random splitting point when considering an optimal split. (3) The gradient boosting model is an ensemble model that uses the boosting technique to combine a sequence of weak decision trees (Friedman, 2001). Each tree in the gradient boosting fits the residuals from the previous tree. Thus, the errors of the previous tree are minimized, and the overall accuracy and robustness of the model are considerably improved. (4) Extreme gradient boosting is an efficient and scalable implementation of the gradient boosting model for sparse data with billions of examples (Chen and Guestrin, 2016). (5) Extremely randomized trees are another model to improve the performance of decision trees by generating diverse ensembles (Geurts et al., 2006). The main idea of this model is to inject randomness into the training process by selecting the best splitting attribute from a random subset of features. However, in contrast to the random forest, the bootstrap instances procedures are implemented by extremely randomized trees. We provide specific explanations for each ML model in the Supplementary material.

Because of the computational burdens of nonparametric models, random search strategies with cross-validation are implemented for the parameter optimization steps. The parameters of decision tree classification, such as the maximum depth of the tree and the minimum number of samples required to split an internal node, are similar to those of regression. The Gini index and entropy are used to measure the quality of a split in classification. Moreover, a large number of parameters of random forest, gradient boosting, extreme gradient boosting, and extremely randomized trees are similar to the parameters of decision tree classification. However, several special parameters can significantly influence performance. Specifically, the number of trees is the most important parameter of random forest classification. The necessary parameters of gradient boosting classification include the loss function to binomial and multinomial deviance, the function to measure the quality of a split, the function to measure the quality of a split, and the number of boosting stages. The parameters of extreme gradient boosting are similar to those of gradient boosting. Its computational speed is faster because it has an option for the number of parallel trees constructed during each iteration. An important parameter of extremely randomized trees is the number of trees in the forest, and the bootstrapping technique is not used to build each tree.

Simple parametric models, such as logistic regression and naïve Bayes, were also included in the classification models. (1) Logistic regression is a standard model for building prediction models for classification. Due to the high-dimensional problems of multiple areas (Bühlmann and van de Geer, 2011), ridge and Lasso penalties are added to penalized logistic modeling for the feature selection step. This model has been applied for the analysis of genetic datasets to select a subset of genes that can provide more accurate diagnostic methods (Liao and Chin, 2007; Wu et al., 2009). (2) Naïve Bayes is a classification model that refers to constructing a Bayesian probabilistic model to assign a posterior class probability to each sample (McCallum and Nigam, 1998). The important assumption of this model is that the features constituting the sample are conditionally independent given the class. The naïve Bayes model is fast, easy to implement, and relatively effective for the classification of biological datasets (Yousef et al., 2007). The grid search strategy with cross-validation is implemented for the parameter optimization steps. The parameter in logistic classification is an elastic net mixing parameter to control the combination of the L1 and L2 regularization. The naïve Bayes classification parameter is an additive (Laplace/Lidstone) smoothing parameter.

To evaluate the decoding performance, three main criteria were compared across tested models: ‘precision’ is defined as the number of true positives over the number of true positives plus the number of false positives, ‘recall’ is defined as the number of true positives over the number of true positives plus the number of false-negatives, and the ‘F1 score’ is defined as the harmonic mean of precision and recall. Figure 1 depicts the steps for searching for important features using FVS, the application of each ML model, and how these computations are appropriately decomposed in a parallel computation manner. All parallel computations were run on a workstation computer (Intel Xeon Gold 6230 Processor 2.10 GHz × 2, 40 cores, 2 threads per core, 128 Gb RAM) under Ubuntu 20.04.1 LTS.

### 2.4. Neuroimaging data samples

To examine the feasibility of the proposed pipeline in neuroimaging, we acquired high-resolution structural MRI scans of a large number of healthy subjects from the Human Connectome Project (HCP). This dataset includes 1,113 samples. The dataset had four age ranges: 22–25, 26–30, 31–35, and more than 36 years; 507 males and 606 females. The structural images were segmented into gray matter, white matter, and cerebrospinal fluid and normalized (1×1×1 voxel size) into a template space using standard parameters implemented in the Computational Neuroanatomy Toolbox (CAT12). During the segmentation process, CAT12 implemented an automated parcellation of the gray matter to extract the gray matter volume in native space from 246 cortical and subcortical brain regions according to neuroanatomical landmarks based on the Brainnetome Atlas^1^ (Fan et al., 2016). CAT12 was also used to estimate individual values of the total intracranial volume (TIV), which was included as a covariate of no interest for the classification and regression models. Notably, the pipeline technically works for the whole-brain voxel-based dataset; however, these segmented data were used for simplicity.

Here, we provide a use case example to identify the best model to predict the target variable. More specifically, the gray matter volume data from 246 Brainnetome regions were selected as target features to predict the age and sex of participants using regression and classification models, respectively.

### 2.5. Package structure

Our framework includes two core modules: automatic ML models and FVS algorithm for regression and classification. First, ML models and the FVS algorithm were implemented using Python programming to optimize the parallel computations that could significantly reduce the computation time. The scikit-learn library in Python was used to implement core computational techniques for the random forest classifier. Our Python package to implement the proposed model is available on GitHub.^2^ In general, each user creates a short script of regression or classification that contains (1) automatic ML models for the input dataset and (2) the FVS algorithm combined with the best ML model in step (1). For example, the script for regression after controlling the effects of variables such as the total intracranial volume (TIV) is short.

>>> from Auto_ML_Regression import AutoML_Regression

>>> from FVS_Regression import AutoML_FVS_Regression

>>> AutoML_Regression.fit(X_train, y_train, X_test, y_test)

This function runs 11 ML regression models to select the best model for the input dataset. The output of this function is a table that shows the rank of performances of 11 ML regression models based on their performance.

>>> AutoML_FVS_Regression.fit(X_train, y_train, X_test, y_test,

model = “LassoLars,” n_selected_features = 100)

After selecting the best ML model, the user implements the function that runs the FVS algorithm to identify an important group of ROIs. For example, the LassoLars model is the best model with the smallest value of MSE in our dataset. Thus, we want to combine the LassoLars model with the FVS algorithm, and the maximum number of features that we want to set is 100. In this case, we define a model as “LassoLars” and ‘n_selected_features’ at 100. The details of the parameters and outputs of all functions in our package are provided in the README.md file on GitHub. Table 1 shows the main functions of our package.

**Table 1:** An overview of the main functions in the FVSdecoder package.

Function	Purpose	Output
Functions from AutoML_Regression		
fit()	Automatic select the best model out of 11 ML regression models	A table shows a rank of performances of 11 ML regression
evaluate_regression()	Show the performance of ML regression	A table shows the MSE and Spearman correlation
Functions from AutoML_Classification		
fit()	Automatic select the best model out of 9 ML classification models	A table shows a rank of performances of 9 ML classification
evaluate_regression()	Show the performance of ML classification	A table shows accuracy, precision, recall, and F1 score
Functions from AutoML_FVS_Regression		
fit()	Combine forward variable selection (FVS) with 11 ML regression models	A table shows the rank of performances of ML regression for a number of features. A table shows a number of selected features
Functions from AutoML_FVS_Classification		
fit()	Combine forward variable selection (FVS) with 9 ML classification models	A table shows the rank of performances of ML classification for a number of features. A table shows a number of selected features

## 3. Results

### 3.1. Improved accuracy for regression models to predict age

Table 2 summarizes the MSE values for each ML model with the Boruta algorithm, with and without the FVS algorithm, to predict the age of healthy individuals. For the comparisons without FVS, the best performance and the smallest MSE (MSE = 0.4541) were obtained using the LassoLars regression model. MSE values were normally distributed for all variable selection algorithms (Lilliefors corrected Shapiro–Wilk test all *p* > 0.18).

**Table 2:** Accuracies of the ML models as assessed by MSE to predict age.

Model	MSE without FVS (#ROIs)	MSE with Boruta (#ROIs)	MSE with FVS (#ROIs)
LassoLar	0.4541 (246)	0.4023 (62)	0.3686 (54)
Random forest	0.4807 (246)	0.4156 (75)	0.3722 (63)
Gaussian process	0.4855 (246)	0.4379 (83)	0.3895 (78)
Ridge	0.4900 (246)	0.4418 (79)	0.3928 (81)
Elastic net	0.4909 (246)	0.4653 (77)	0.4011 (73)
Lars	0.4988 (246)	0.4728 (61)	0.4171 (68)
Lasso	0.5034 (246)	0.4831 (67)	0.4265 (59)
Kernel ridge	0.5061 (246)	0.4927 (82)	0.4402 (71)
Multitask lasso	0.5341 (246)	0.5156 (66)	0.4578 (52)
Decision tree	0.5669 (246)	0.5318 (71)	0.4669 (76)
Stochastic gradient descent	0.5687 (246)	0.5475 (78)	0.5000 (80)

FVS, forward variable selection algorithm; MSE, mean squared error; ROI, region of interest. Entries are sorted in order of ascending MSE values.

**Figure 2:**
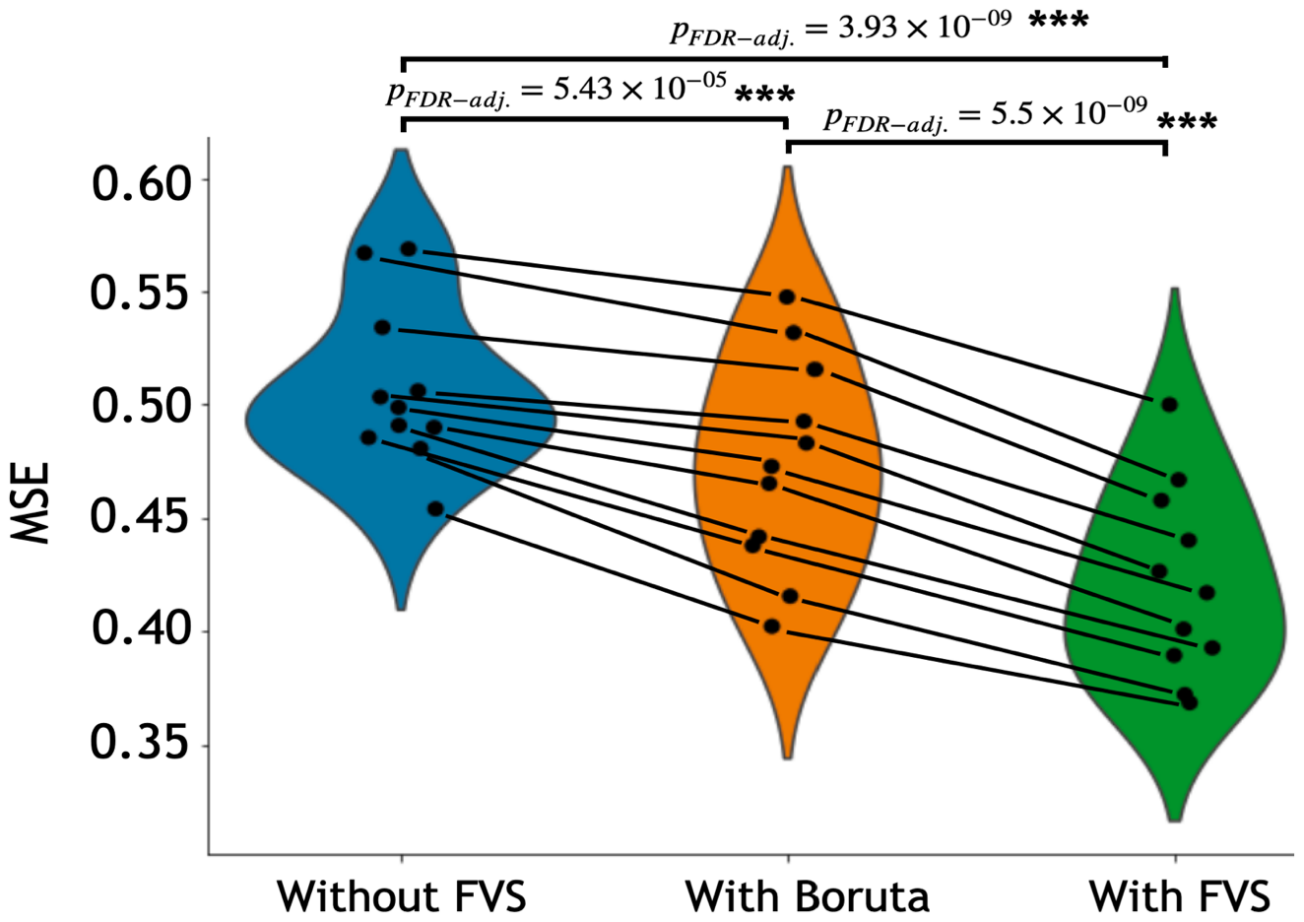
Performance comparison of 11 regression models with Boruta algorithm, with and without forward variable selection (FVS) to predict age, controlling for total intracranial volume (TIV). Left (blue): 11 regression models without the FVS algorithm. Middle (orange): 11 regression models on a subset of brain regions selected with the Boruta algorithm. Right (green): 11 regression models on a subset of brain regions selected with the FVS algorithm. *P* values were calculated using one-way repeated measures ANOVA tests with Benjamini–Hochberg correction for multiple comparisons for 11 pairs of models. *p < 0.05, **p < 0.01, ***p < 0.001.

The variable selection model had a very strong effect (*F*_2,20_ = 225.521; *p* < 0.001; partial *η*^2^ = 0.958). *Post Hoc* analyses revealed that across the 11 models, Boruta improved the decoding accuracy over the ‘without FVS’ (*p* < 0.001; Cohen’s *d* = 0.815), as was expected. Beyond the Boruta algorithm, the use of the FVS algorithm significantly improved the performance against both ‘without FVS’ (*p* < 0.001; Cohen’s *d* = 2.216) and ‘with Boruta’ (*p* < 0.001; Cohen’s *d* = 1.177) with very large effect size (see Figure 2). Notably, it is essential to consider the computational cost (see Supplementary Table S1 for the computational cost of each model and algorithm). In terms of decoding accuracy, ‘random forest (second best)’ and ‘Gaussian process (third best)’ models *without* variable selection comparatively were as good as the LassoLars regression model. However, the computational costs of the random forest (CPU time = 242.0 s without FVS) and Gaussian process (CPU time = 80.2 s without FVS) models were more excessive because the parameters were more complex than those of LassoLars regression (CPU time = 20.3 s without FVS). The other models, such as ridge, elastic net, and Lars regression, had faster computations but did not satisfy reasonable performance. Therefore, we focused on the LassoLars regression model for the next step of the analysis.

**Figure 3:**
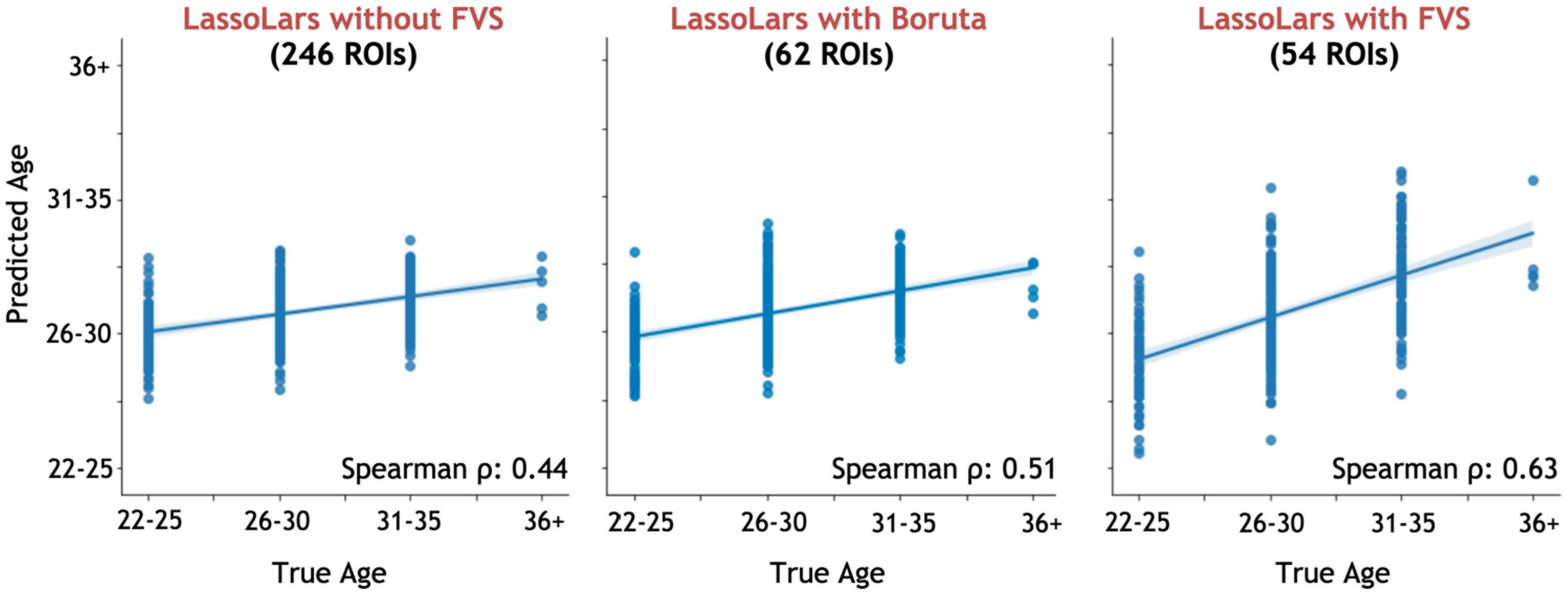
Performance comparison of LassoLar regression with Boruta algorithm, with and without the forward variable selection (FVS) algorithm to predict age, controlling for the effects of total intracranial volume (TIV). Left panel: LassoLar regression with all of brain regions (MSE = 0.45, Spearman *ρ* = 0.44, *p* = 0.064). Middle panel: LassoLar regression on a subset of brain regions selected with the Boruta algorithm (MSE = 0.4, Spearman *ρ* = 0.51, *p* < 0.0001). Right panel: LassoLar regression on a subset of brain regions selected with the FVS algorithm (MSE = 0.36, Spearman *ρ* = 0.63, *p* < 0.0001). Predicted age data are plotted as a function of the true score. The blue lines and blue shades represent a linear regression line with a confidence interval.

Among all model comparisons, 54 out of 246 brain regions were identified with a Spearman correlation coefficient of 0.63 (*p* < 0.0001, Figure 3) using the FVS-supported LassoLar regression model. The Boruta-supported LassoLar regression model showed a comparable accuracy with a Spearman correlation coefficient of 0.51 (*p* < 0.0001). Comparing the FVS and Boruta algorithms, both algorithms commonly selected 20 ROIs, such as the thalamus, hippocampus, amygdala, orbital gyrus, and superior frontal gyrus (see Supplementary Table S3). However, there were several differences in the selected features. While the ‘FVS-supported LassoLar’ model uniquely selected several ROIs such as parahippocampal gyrus, insula gyrus, basal ganglia, and angular gyrus (see Supplementary Table S4), the ‘Boruta-supported LassoLar’ model selected inferior parietal gyrus, inferior temporal gyrus, inferior frontal gyrus (see Supplementary Table S5). Focusing only on the three best FVS-supported models (namely, LassoLar, Random Forest, and Gaussian Process), several commonly selected ROIs are thought to be important for age: thalamus, hippocampus, and insula cortex. As was the case for the differences in FVS and Boruta algorithms, different ROIs were selected by each model (see Supplementary Tables S4, S5 for the details).

**Figure 4:**
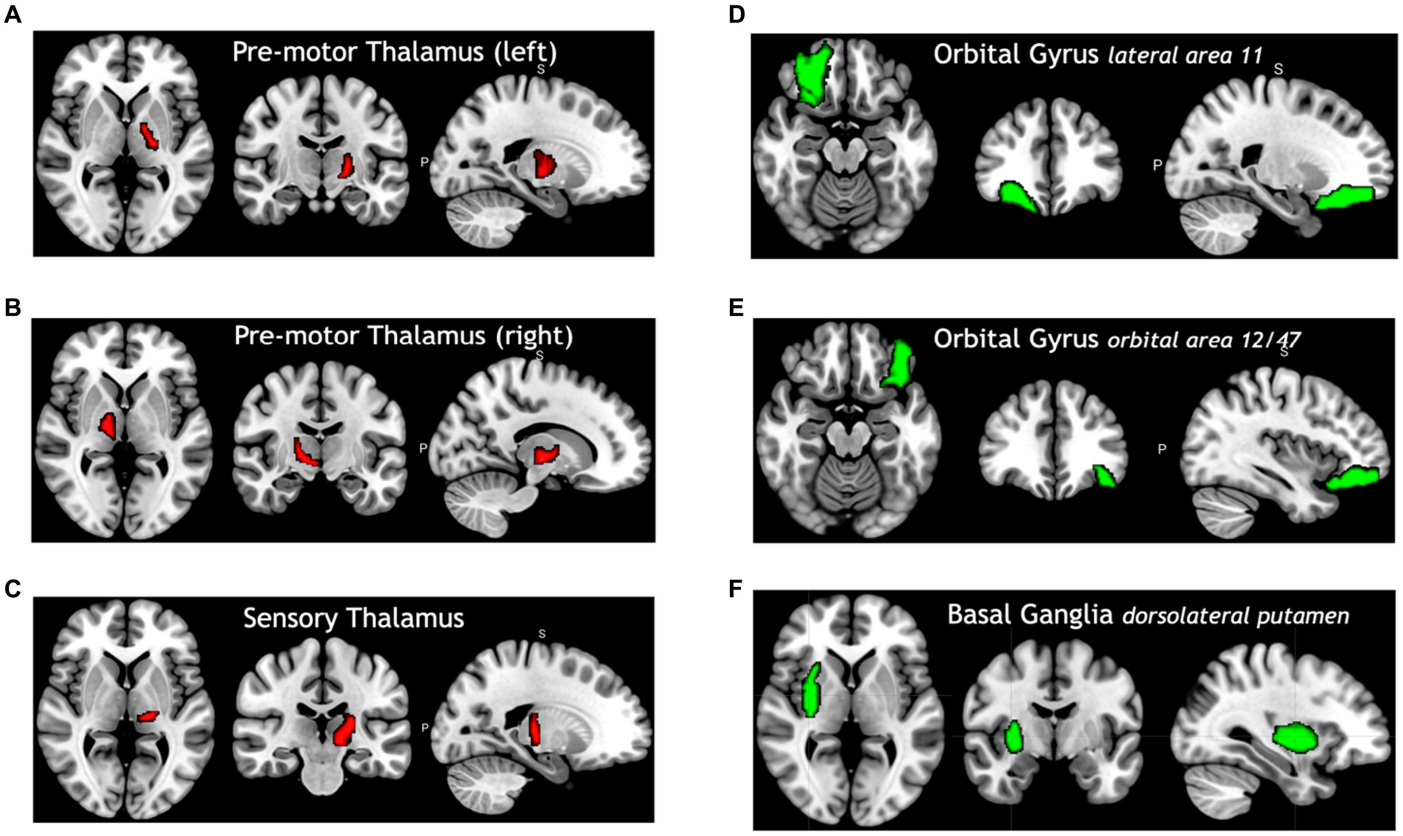
Selected brain regions significantly associated with age. The red color denotes a positive correlation with age; the green color denotes a negative correlation with age. **(A)** Premotor thalamus (left), **(B)** premotor thalamus (right), **(C)** sensory thalamus (left), **(D)** orbital gyrus lateral area 11, **(E)** orbital gyrus orbital area 12/47, **(F)** basal ganglia dorsolateral putamen.

Figure 4 shows selected brain regions identified by the FVS-supported LassoLars model. As it turned out, these results were consistent with previous reports on the association of brain regions with age. For example, the thalamus plays a critical role in the coordination of information flow in the brain, mediating communication and integrating many processes, including memory, attention, and perception. Thus, age-related cognitive capability could be associated with micro- and macrostructural alterations in the thalamus. A number of previous studies have shown that increasing age significantly influences the changes in the thalamus (Good et al., 2001; Hutton et al., 2009).

### 3.2. Improved accuracy for classification models to identify sex

**Figure 5:**
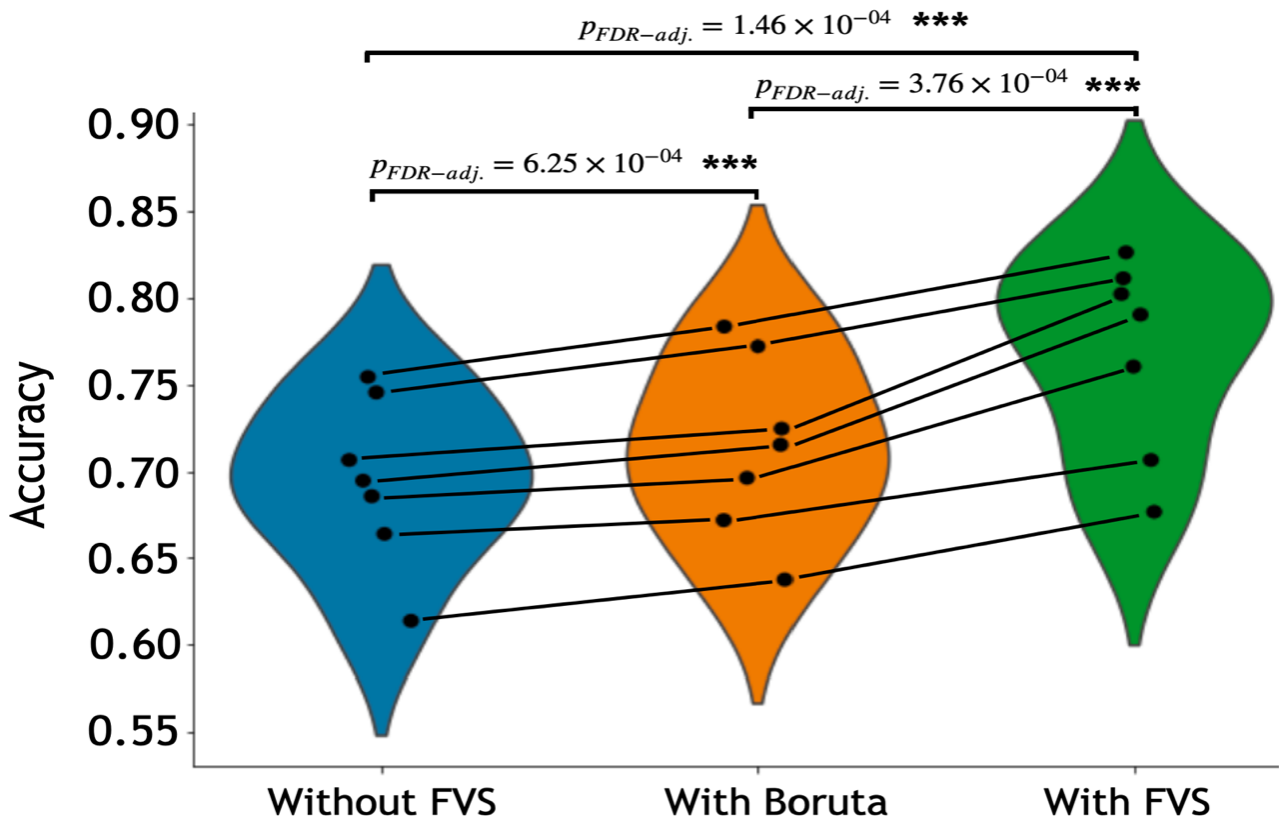
Performance comparison of 7 classification models with Boruta algorithm, with and without the forward variable selection (FVS) algorithm to classify male and female groups, controlling for the effects of total intracranial volume (TIV). Left (blue): 7 classification models without the FVS algorithm. Middle (orange): 7 classification models on a subset of brain regions selected by the Boruta algorithm. Right (green): 7 classification models on a subset of brain regions selected by the FVS algorithm. *P* values were calculated using one-way repeated measures ANOVA tests with Benjamini–Hochberg correction for multiple comparisons. *p < 0.05, **p < 0.01, ***p < 0.001.

Table 3 summarizes the accuracies for each ML model with the Boruta algorithm, with and without the FVS algorithm that classifies the male and female groups. The best performance among the comparisons without the FVS algorithm, with the highest accuracy of 75.44%, was obtained using the random forest classifier. Accuracy values were normally distributed for all variable selection algorithms (Lilliefors corrected Shapiro–Wilk test all *p* > 0.31). There was a very strong effect of the variable selection model (*F*_2,12_ = 79.843; *p* < 0.001; partial *η*^2^ = 0.930). *Post Hoc* analyses revealed that across the 7 models, Boruta improved the decoding accuracy over the ‘without FVS’ (*p* < 0.001; Cohen’s *d* = 0.389) as was expected. Beyond the Boruta algorithm, the use of the FVS algorithm significantly improved the performance against both ‘without FVS’ (*p* < 0.001; Cohen’s *d* = 1.394) and ‘with Boruta’ (*p* < 0.001; Cohen’s *d* = 0.985) with very large effect size (see Figure 5).

**Table 3:** Accuracies of the ML models used to classify the male and female groups.

Model	Accuracy (%) without FVS (#ROIs)	Accuracy (%) with Boruta (#ROIs)	Accuracy (%) with FVS (#ROIs)
Random forest	75.44 (246)	78.35 (73)	82.63 (87)
Extreme gradient boosting	74.55 (246)	77.21 (110)	81.13 (92)
Logistic regression with the absolute norm L1	70.65 (246)	72.46 (88)	80.23 (98)
Gradient boosting	69.46 (246)	71.53 (81)	79.04 (76)
Extremely randomized trees	68.56 (246)	69.62 (102)	76.04 (81)
Decision tree	66.39 (246)	67.18 (77)	70.65 (68)
Naïve bayes	61.37 (246)	63.75 (64)	67.76 (53)

FVS, forward variable selection algorithm; ROI, region of interest. Entries are sorted in order of descending accuracy values.

Notably, it is essential to consider the computational cost (see Supplementary Table S2 for the computational cost of each model and algorithm). In terms of decoding accuracy, ‘extreme gradient boosting (second best)’ models (74.55%) *without* variable selection comparatively were as good as the random forest model (75.44%, see Table 3). However, the computational costs of the extreme gradient boosting model (CPU time = 270.0 s without FVS) model were more expensive because the parameters were more complex than those of the random forest classifier (CPU time = 150.0 s without FVS). Additionally, logistic regression with the absolute norm L1 achieved a fairly comparable performance (70.65%) to the extreme gradient boosting classifier (74.55%), while the computational time of logistic regression was considerably shorter (CPU time = 19.0 s without FVS) than that of extreme gradient boosting classifier (CPU time = 270.0 s without FVS). Conversely, the extremely randomized trees and naïve Bayes models had poor performances with low accuracy values (68.56 and 61.37%).

**Figure 6:**
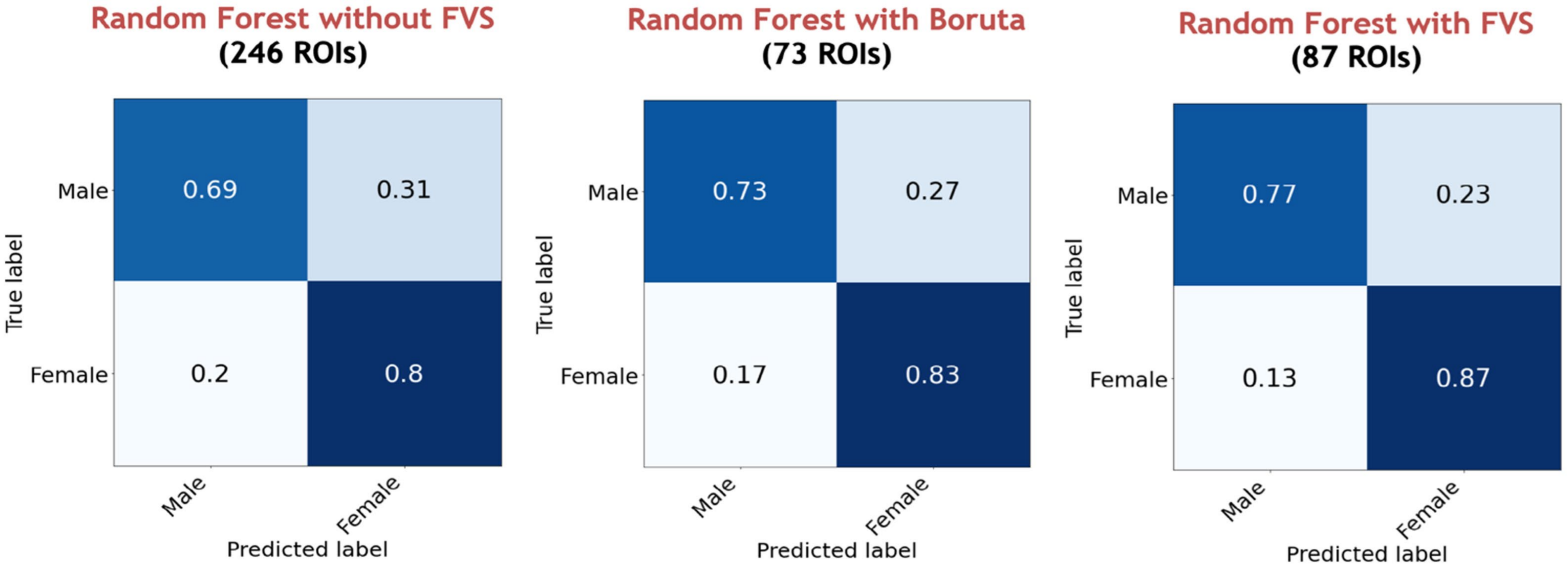
Performance comparison of the random forest classifier with Boruta algorithm, with and without the forward variable selection (FVS) algorithm to classify two groups, controlling for the effects of total intracranial volume (TIV). Left panel: random forest classifier analysis with all of brain regions. Middle panel: random forest classifier on a subset of brain regions selected by the Boruta algorithm. Right panel: random forest classifier on a subset of brain regions selected by the FVS algorithm.

**Figure 7:**
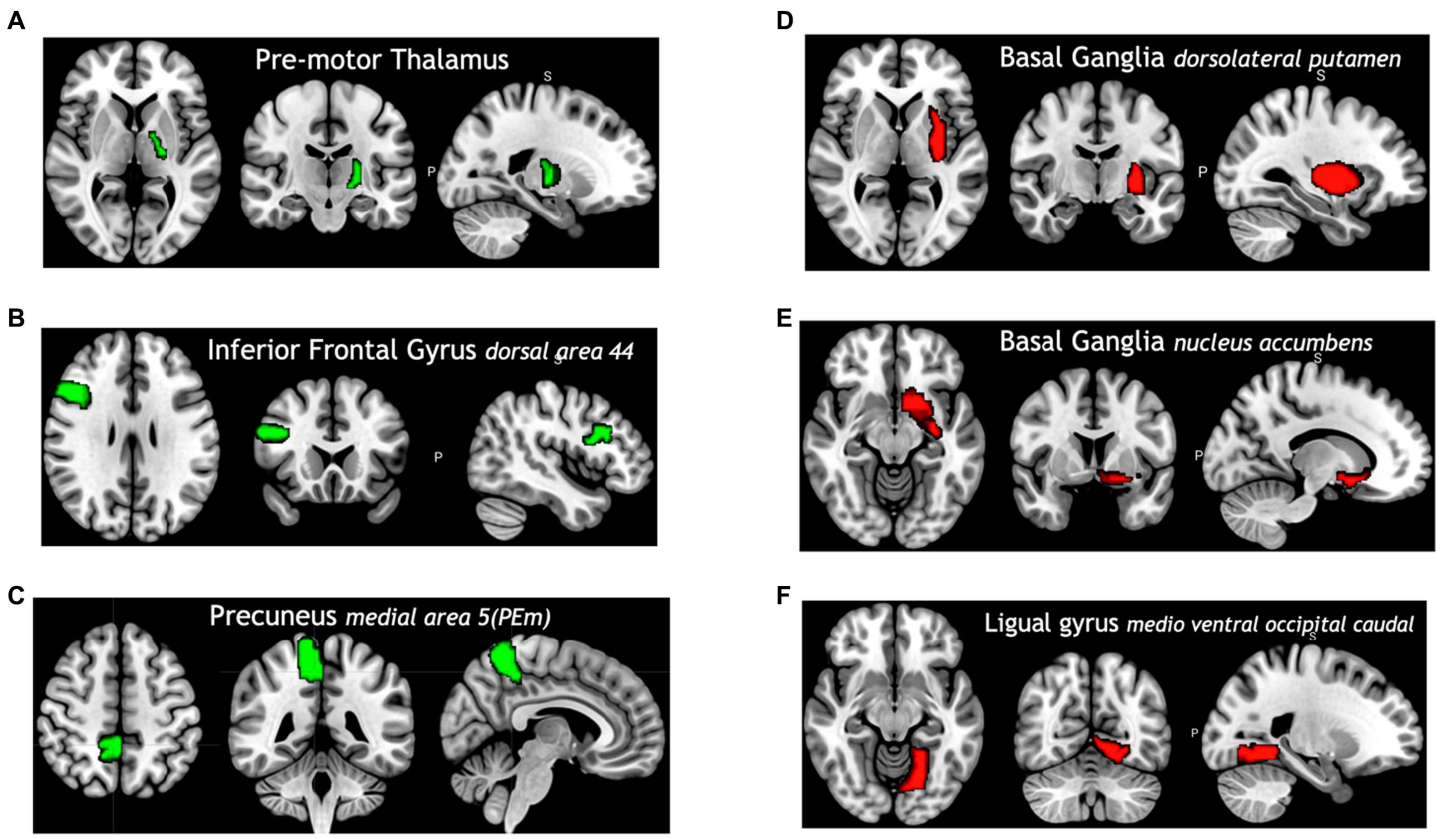
Selected brain regions identified as predictors of the sex categories (male and female). The red color denotes male predicting volume > female predicting volume; the green color denotes male predicting volume < female predicting volume. **(A)** Premotor thalamus, **(B)** inferior frontal gyrus dorsal area 44, **(C)** precuneus medial area 5 (PEm), **(D)** basal ganglia dorsolateral putamen, **(E)** basal ganglia nucleus accumbens, **(F)** ligual gyrus medio ventral occipital caudal.

Among all model comparisons, Figure 6 shows that 87 out of 246 brain regions were identified, and the accuracy improved to 82.63% using the FVS-supported random forest classifier. Females were identified with an accuracy of 87%, and males were identified with an accuracy of 77%. The Boruta-supported random forest classifier identified 73 out of 246 brain regions and achieved an accuracy of 78.35%. Figure 7 shows that the selected brain regions, such as the thalamus, inferior frontal gyrus, precuneus, and basal ganglia, were mapped on the Brainnetome Atlas. These brain regions were identified by our model and were consistent with previous reports. For example, a number of studies showed that females had significantly greater volumes in the inferior frontal gyrus, thalamus, and precuneus. Conversely, males had significantly greater volumes in the basal ganglia and lingual gyrus. Comparing the FVS and Boruta algorithms, both algorithms commonly selected 36 ROIs, such as the thalamus, inferior frontal gyrus, inferior parietal gyrus, basal ganglia, and middle frontal gyrus. However, there were several differences in the selected features (see Supplementary Table S6). While the ‘FVS-supported Random Forest’ model uniquely selected several ROIs such as superior frontal gyrus, superior parietal gyrus, fusiform gyrus, and cingulate (see Supplementary Table S7), the ‘Boruta-supported Random Forest’ model selected orbital gyrus, postcentral gyrus, lateral occipital gyrus (see Supplementary Table S8). Focusing only on the two best FVS-supported models (namely, Random Forest and extreme gradient boosting), several commonly selected ROIs are thought to be important for sex: thalamus, cingulate, and inferior frontal gyrus. As was the case for the differences in FVS and Boruta algorithms, different ROIs were selected by each model (see Supplementary Tables S7, S8 for details).

## 4. Discussion

In this study, a parallelized FVS toolbox is developed to provide optimized decoding of neuroimaging data samples. Our toolbox can be used to propose the best ML model for user’s input data and identify a small group of important features that significantly improve the performance of the ML model. We have demonstrated that the toolbox is feasible for region of interest (ROI) data without revising the model types (parametric or nonparametric) and parameter settings, suggesting that this toolbox is generalizable and could potentially be used to train multiple types of neuroimaging data without modification. Given previous use cases of the ML model that have been established in genetic studies using the FVS algorithm (Dang and Kishino, 2022), we have extended the FVS algorithm and the toolbox has been created for neuroimaging studies. To examine the feasibility of our ML pipelines, sample neuroimaging data were acquired from the HCP database. As case samples, we compared the accuracies (predictability) of the classical ML models with and without the FVS algorithm.

We tested the performances of several ML models by analyzing large structural MRI datasets with a large number of variables (246 brain regions). An easy-to-use computational package may help novel data scientists in neuroimaging research and advance the research by identifying accurate features relevant to questions of interest.

### 4.1. Comparison against existing methods

The proposed method presents the following advantages compared with the previous methods. First, neuroscientists could avoid decision uncertainties when considering or choosing the most appropriate model for their own datasets. In our proposed method, users only provide the input data and decide whether to run the proposed ML pipeline for either classification or regression based on their purpose of analysis. The automatic algorithm will rank the ML models and recommend the best model for the user’s dataset. In this study, the results showed that random forest was the most accurate model for classification. Random forest is an ML model that is based on combining multiple decision trees by random selection of samples. Therefore, random forest overcomes the problem of overfitting decision trees, which can result in a better fitting of the model (Ghose et al., 2012; Mitra et al., 2014; Sarica et al., 2017; Zhu et al., 2018). In the regression task, the best performance for predicting the age of healthy individuals was obtained using the LassoLar model. The performance of random forest was ranked second (Smith et al., 2013; Jog et al., 2017; Dimitriadis et al., 2018). In the second step, the FVS algorithm was used to select a feature (e.g., ROI) that improves the accuracies of ML classification models or reduces the MSEs of ML regression models at each iteration. This procedure was stopped if the performance of the ML model reached a maximization. The FVS algorithm attempts to identify a minimal core set of brain regions that can provide insights into brain functions. The results showed that the performances of all ML models in classification and regression were significantly improved after applying the FVS algorithm. For example, the FVS algorithms identified 87 ROI features that improved the accuracy of the random forest classifier from 75.44 to 82.63%.

### 4.2. Advantages of FVS

In the regression model, the option with FVS significantly outperformed the option without FVS and with the Boruta algorithm (Figures 2, 5, respectively). For the regression model to identify age, the LassoLars model was selected as the best model, and 54 regions to account for age were identified. In brief, this finding suggests that the thalamus and orbital gyrus are significantly associated with age-related changes. A previous study found that a general linear model identified age-related changes in terms of gray matter density (Tisserand et al., 2004). The prefrontal cortex (PFC), the (medial) temporal lobe, and the posterior parietal cortex showed the greatest differences in gray matter density.

For the classification model to identify regions that account for sex, the random forest model was determined to be the best model. This result suggests that the inferior frontal gyrus, thalamus, and precuneus regions may contribute to identifying sex. A previous study (Xu et al., 2000) suggested that the posterior right frontal lobe, right temporal lobe, left basal ganglia, parietal lobe, and cerebellum regions may contribute to identifying differences between males and females.

### 4.3. Limitations

Although it was apparent that the FVS algorithm robustly and significantly improved the accuracy for both classification and regression models, the downside of this model is the computation time to apply nearly all possible pairs to consider all features (up to the specified number of pairs specified by the user). To compensate for the issue of time, the parallel computing pipelines implemented in our toolbox effectively minimize and compensate for the computational time.

While the FVS algorithm significantly improves the performances of the ML models, the computational burden of the FVS algorithm is still a difficult challenge for personal computers. Even if we apply the parallel computational techniques to overcome large-scale problems in the FVS algorithm, a high-performance computer, but not on a low-spec computer, is necessary to efficiently run our proposed tool. Although the computational speed could be improved, based on the material efficiency aspects of personal computers, implementing our strategy would still not be possible. In the future, we may implement a new method (Xing et al., 2016) that can balance high-speed computation and material efficiency.

Furthermore, instead of a voxel-based approach, atlas-based analyses were performed in this study for demonstrational purposes. One could apply the proposed method to voxel-based datasets in future studies. It has been shown that the differential outcomes between voxel-based and atlas-based analyses to identify structural brain alterations between groups (Seyedi et al., 2020). Therefore, the reported observations in this study may differ from those using a voxel-based approach. The best model for determining age in our study, for example, identified several brain regions reported to be associated with age (i.e., thalamus, hippocampus, amygdala, orbital gyrus, and superior frontal gyrus) as reported in the previous works (Good et al., 2001; Zhou et al., 2022). However, certain brain regions reported in these studies were not chosen via our approach; these include the postcentral gyrus, superior temporal gyrus, brainstem, medial frontal cortex, middle temporal gyrus, middle frontal gyrus, and cerebellum. The same holds true for the classification models for sex. Although the amygdala, precuneus, cerebellum, parietal operculum cortex, and orbital cortex were reported as significant regions to classify sex, we only found a limited overlap such as the thalamus, inferior frontal gyrus, inferior parietal gyrus, basal ganglia (Ruigrok et al., 2014).

Although direct comparisons of decoding accuracies were made, it would be important to be aware that the oFVSD, or ML in general, may not necessarily identify the same brain regions as previous studies. While our data-driven feature selection approach certainly benefits from blind-folded neural decoding, on the other hand, the FVS approach is rather greedy, and it may lead to local minimas and may not necessarily reflect scientific rigorousness based on existing evidence. Our toolbox may further practically and logically benefit from human supervision based on existing literature by restricting target features to scientifically validated brain regions of interest (Chu et al., 2012). That said, users of the oFVSD need to carefully interpret the outcome due to the pitfalls of the data-driven ML approach that this toolbox may offer.

### 4.4. Computational time

The high-dimensional problems of these datasets will result in more difficult challenges for the FVS algorithms. The FVS algorithm was applied to analyze the 16S rRNA sequencing microbiome datasets, where the number of features was huge (approximately 30,000 features) in our previous study (Dang and Kishino, 2022). To reduce the computational burden of the FVS algorithm, some prescreening algorithms [such as the Boruta algorithm (Kursa and Rudnicki, 2010) and Laplacian score (He et al., 2005)] were proposed to detect all strongly and weakly relevant features to reduce the considerable data dimensionality. With the initial prescreening pipeline, the computational time of the FVS algorithm could be significantly decreased from days to hours (Dang and Kishino, 2022). The current pipeline uses a fixed prescreening model. Therefore, additional considerations of this strategy may be necessary if the number of features becomes very large to apply a rigorous search method such as our approach.

## 5. Conclusion

The use of neuroimaging data to train ML models has a significant potential for identifying brain regions whose structure and activities may contain information predictive of physical phenotypes, mental states, and pathological conditions. However, an overwhelmingly large number of ML models exist, which may increase the difficulties for those unfamiliar with mathematical theories. Moreover, the high dimensionality of neuroimaging data negatively impacts the power of ML models to discover hidden information in the selected neural resources. Furthermore, researchers are often challenged with time-consuming computations to identify neural substrates, a variety of neuroscientific discoveries, and the development of novel therapeutic interventions. In this study, we proposed a novel procedure that not only automatically selects the best ML model for specific neuroimaging data but also identifies a group of brain regions that substantially improve the performance in terms of high-speed computation and high accuracy. This powerful decoding tool may be applicable to a variety of neuroimaging modalities.

## Data availability statement

The original contributions presented in the study are included in the article/Supplementary material, further inquiries can be directed to the corresponding author.

## Author contributions

TD: Conceptualization, Formal analysis, Investigation, Methodology, Software, Visualization, Writing – original draft, Writing – review & editing. AF: Formal analysis, Writing – original draft, Writing – review & editing. MM: Conceptualization, Data curation, Funding acquisition, Project administration, Supervision, Visualization, Writing – original draft, Writing – review & editing.

## Funding

The author(s) declare financial support was received for the research, authorship, and/or publication of this article. This work was supported by JSPS KAKENHI (Grant Number JP21J21850), the JST COI Grant Numbers: (JPMJCE1311 and JPMJCA2208), and the Moonshot R&D Goal 9 (JPMJMS2296).

## Conflict of interest

The authors declare that the research was conducted in the absence of any commercial or financial relationships that could be construed as a potential conflict of interest.

## Publisher’s note

All claims expressed in this article are solely those of the authors and do not necessarily represent those of their affiliated organizations, or those of the publisher, the editors and the reviewers. Any product that may be evaluated in this article, or claim that may be made by its manufacturer, is not guaranteed or endorsed by the publisher.

## Footnotes

^1^https://atlas.brainnetome.org/bnatlas.html

^2^https://github.com/tungtokyo1108/FVS_decoder
