# Supplementary material for: oFVSD: a Python package of optimized forward variable selection decoder for high-dimensional neuroimaging data
Source: Front Neuroinform. 2023 Sep 26;17:1266713. doi: 10.3389/fninf.2023.1266713 (PMC10566623; doi:10.3389/fninf.2023.1266713)
Supplement: Supplementary file 1 [file Data_Sheet_1.pdf]

frontiers

Supplementary Material

oFVSD: A Python package of optimized forward variable selection decoder for high-dimensional neuroimaging data

Tung Dang[1,2], Alan S. R. Fermin[1], Maro G. Machizawa[1*]

[1] Center for Brain, Mind, and KANSEI Sciences Research, Hiroshima University, Hiroshima, 734-8551 Japan

[2] Graduate School of Agricultural and Life Sciences, The University of Tokyo, Tokyo, Japan

*** Correspondence:** Corresponding Author
machizawa@hiroshima-u.ac.jp

Description of machine learning models

In this appendix we provide a theoretical overview for all models evaluated, together with technical details on their implementation.

1 Ridge regression

There are response variable $\boldsymbol{y} = \{y_1, \dots, y_i\}_{i=1}^{N}$ and the number of independent features $\boldsymbol{x} = \{x_{i1}, x_{i2}, \dots, x_{ip}\}_{p=1}^{P}$ where N is a number of samples and P is a number of features. The classical way is the ordinary least square (OLS) algorithm which minimizes the squared loss:

$$\sum_{i=1}^{N} (y_i - \sum_{p=1}^{P} \beta_p \, x_{ip})^2 \qquad \textbf{(1)}$$

Because the number of features (i.e. number of voxels, cortical vertices or regions) is much larger than the number of samples (p >> n) in high-dimensional datasets, the variance of the estimate *w* by OLS may be large and thus the estimate is not reliable. Ridge regression (Hastie et al., 2009) can reduce the variance by penalizing the norm of the linear transform and minimizes the following cost:

$$J(\boldsymbol{\beta}) = \sum_{i=1}^{N} (y_i - \sum_{p=1}^{P} \beta_p \, x_{ip})^2 + \lambda \sum_{p=1}^{P} \beta_p^2 \qquad \textbf{(2)}$$

Where λ is the regularization parameter to controls the trade-off between the bias and variance of the estimate. In practice, one can use cross-validation (Hastie et al., 2009) to find the optimal regularization parameter.

2 Lasso regression

Lasso regression replaces squares by absolute values and minimizes the following cost (Hastie et al., 2009; Tibshirani, 1996):

$$J(\boldsymbol{\beta}) = \sum_{i=1}^{N}(y_i - \sum_{p=1}^{P} \beta_p\, x_{ip})^2 + \lambda \sum_{p=1}^{P} |\beta_p| \tag{3}$$

Ridge regression scales the coefficients by a constant factor, whereas the lasso translates by a constant factor, truncating at 0.

3 Elastic Net

The elastic net (EN) method consists of the addition of an L2 penalty to the lasso penalty one to obtain a linear combination of these two norms (Hastie et al., 2009; Zou and Hastie, 2005). The objective is to inherit both the stability of ridge regression, for highly correlated features and the variable selection property of the lasso:

$$J(\boldsymbol{\beta}) = \sum_{i=1}^{N}(y_i - \sum_{p=1}^{P} \beta_p\, x_{ip})^2 + \lambda_2 \sum_{p=1}^{P} \beta_p^2 + \lambda_1 \sum_{p=1}^{P} |\beta_p|$$

4 Least angle regression (LAR)

LAR is a computational efficient variant of linear regression with an L1-regularization term (Efron et al., 2004; Hastie et al., 2009). Like the traditional forward selection method, LAR starts with a zero vector as the initial solution (i.e. no active variables), and adds a new predictor variable (i.e. an active variable) at every step. LAR avoids the computational burden of the forward selection method in calculating the coefficients of the active variables.

Specifically, the LAR model includes some main steps:

1. LAR starts with the residual $\boldsymbol{r} = \boldsymbol{y} - \boldsymbol{X\beta}$; $\beta_1 = \beta_2 = \cdots = \beta_p = 0$.
2. LAR identifies the predictor that correlates most with $\boldsymbol{r}$ (i.e. one that forms the least angle with the residual vector), say x_{im} and add x_{im} to the active set.
3. LAR moves β_m from 0 towards its least-squares coefficient $\langle x_{im}, \boldsymbol{r} \rangle$ until some other competitor x_{ik} has as much correlation with the current residual.
4. The solution β_m and β_k is updated along the direction defined by their joint least squares coefficient of the current residual on (x_{im}, x_{ik}), until the residuals become equally correlated with another predictor x_{il} which is outside the active set.
5. x_{il} is added to the active set, and the process is repeated until completion or until a desired number of active variables is reached.

5 The LAR–Lasso combination

Suppose **A** is the active set of variables at some stage in the LAR model, tied in their absolute inner-product with the current residuals $\boldsymbol{r} = \boldsymbol{y} - \boldsymbol{X\beta}$. We can express this as (Hastie et al., 2009)

$$x_p^T(\boldsymbol{y} - \boldsymbol{X\beta}) = \gamma \boldsymbol{s}_p, \forall p \in \boldsymbol{A}$$

Where $\boldsymbol{s}_p \in \{-1, 1\}$ indicates the sign of the inner-product, and γ is the common value. Now consider lasso criterion in equation 3, **B** be the active set of variables in the solution for a given value of λ in equation 3. For these variables $J(\boldsymbol{\beta})$ is differentiable, and the stationarity conditions give

$$x_p^T(\boldsymbol{y} - \boldsymbol{X\beta}) = \lambda\, sign(\beta_p), \forall p \in \mathbf{B}$$

6 Multi-task Lasso regression

N_m, P and M denote the number of samples for the m-th task, the number of features for each input matrix, and the number of tasks, respectively. We assume that all the input matrix in $\{X^m, m = 1, 2, \ldots, M\}$ are having the same dimensionality of features. The multi-task learning with Lasso constraint is given as below (Thung and Wee, 2018; Zhang and Yang, 2022):

$$\min_{\boldsymbol{\beta}} \sum_{m-1}^{M} L(\boldsymbol{X}^m, \boldsymbol{y}^m, \boldsymbol{\beta}^m) + +\lambda \|\boldsymbol{\beta}\|_1 \tag{4}$$

Where $\boldsymbol{\beta} = [\beta^1 \beta^2 \ldots \beta^M]$ and λ is the regularization parameter that controls sparsity in $\boldsymbol{\beta}$

7 Regularized linear models with stochastic gradient descent (SGD)

We rewrite the problem in equation 3 of Lasso regression (Shalev-Shwartz and Tewari, 2011):

$$\min_{\boldsymbol{\beta}} \frac{1}{N} \sum_{i=1}^{N} L(\langle \boldsymbol{\beta}, \boldsymbol{x}_i \rangle, y_i) + \lambda \|\boldsymbol{\beta}\|_1$$

The stochastic coordinate descent model includes main steps:

1. Initialize $\boldsymbol{\beta}$ to be 0
2. At each iteration, we pick a coordinate p uniformly at random from set of features [P] = {1, …, P}
3. The derivative w.r.t the jth feature $g_j = \frac{1}{N} \sum_{i=1}^{N} L'(\langle \boldsymbol{\beta}, \boldsymbol{x}_i \rangle, y_i) x_{i,p}$, where L' is the derivative of the loss function with respect to its first argument.
4. With step size $\boldsymbol{\tau}$, we update $\boldsymbol{\beta}_p \leftarrow s_{\lambda/\tau}(\boldsymbol{\beta}_p - g_j/\tau)$

8 Kernel ridge regression

Kernel ridge regression combines ridge regression with the kernel trick (Vladimir Vovk, 2013). The data is now replaced with the feature vectors: $x_i \rightarrow \Phi_i = \Phi(x_i)$ induced by a kernel where $k(x_i, x_j) = \Phi(x_i)^T \Phi(x_j)$. Here $k(.,.)$ is the kernel function which is typically linear $k(x_i, x_j) = x_i{}^T x_j$, polynomial $k(x_i, x_j) = (x_i{}^T x_j + 1)^d$ or Gaussian $k(x_i, x_j) = exp({}^{-\|x_i - x_j\|^2}/_{\sigma^2})$. Kernel ridge regression minimizes the following cost:

$$J(\boldsymbol{\beta}) = (\boldsymbol{y} - \mathrm{K}\boldsymbol{\beta})^T(\boldsymbol{y} - \mathrm{K}\boldsymbol{\beta}) + \lambda \boldsymbol{\beta}^T \mathrm{K}^T \boldsymbol{\beta}$$

Where K is the kernel matrix $K = \begin{bmatrix} k(x_1, x_1) & \cdots & k(x_1, x_N) \\ \vdots & \ddots & \vdots \\ k(x_N, x_1) & \cdots & k(x_N, x_N) \end{bmatrix}$

9 Decision tree

A decision tree classifies data items by posing a series of question about the features associated with the items. An internal node contains each question, decision trees are grown by adding question nodes, using labeled training examples to guide the choice of questions. Gini index and entropy are two most common measures that are designed to evaluate the degree of inhomogeneity, or impurity in a set of items. Suppose we want to classify items into K classes. In a node m, representing a region R_m with N_m observations, the proportion of class k observations in node m is calculated as follow (Hastie et al., 2009):

$$\widehat{p_{mk}} = \frac{1}{N_m} \sum_{x_i \in R_m} I(y_i = k)$$

Cross-entropy measure is calculated as follow (Hastie et al., 2009):

$$-\sum_{k=1}^{K} \widehat{p_{mk}} \log \widehat{p_{mk}}$$

where the entropy is lowest when a single $\widehat{p_{mk}}$ equals 1 and all others are 0, whereas if all $\widehat{p_{mk}}$ are equal, it is the largest.
Gini index is calculated as follow (Hastie et al., 2009):

$$\sum_{k \neq k'} \widehat{p_{mk}}\, \widehat{p_{mk'}} = \sum_{k=1}^{K} \widehat{p_{mk}}\, (1 - \widehat{p_{mk}})$$

To avoid overfitting the training data, we must prune the tree by deleting nodes. There are some approaches such as minimum description length, keep the balance of the complexity of the tree and its fit to the training data by removing internal nodes.
In case of regression, we have p inputs $\{x_{i1}, x_{i2}, \dots, x_{ip}\}_{p=1}^{P}$ and a response $\boldsymbol{y} = \{y_1, \dots, y_i\}_{i=1}^{N}$. we model the response as a constant c_m in each region (Hastie et al., 2009):

$$f(x) = \sum_{m=1}^{M} c_m\, I(x \in R_m)$$

The greedy model is used to optimate parameters of decision tree regression. There are a number of main steps as follow:

1. Consider a splitting variable j and split point s, and define the pair of half-planes
   - $R_1(j, s) = \{X | X_j \leq s\}$ and $R_2(j, s) = \{X | X_j > s\}$
2. For any choice j and s, the inner minimization is solved by
   - $\widehat{c_1} = average\ (y_i | x_i \in R_1(j, s))$ and $\widehat{c_2} = average\ (y_i | x_i \in R_2(j, s))$
3. The splitting variable j and split point s
   - $\min_{j,s} [\min_{c_1} (y_i - \widehat{c_1})^2 + \min_{c_2} (y_i - \widehat{c_2})^2]$

10 Random forest

In random forest model, a number of decision trees are grown by a randomized tree-building model. Random forest produces a modified training set of equal size by sampling with replacement the training set. Moreover, this model selects randomly subset of the features when considering the question at each node. There are a number of main steps as follow (Hastie et al., 2009):

1. Consider a number of decision trees B. For each decision tree
   - Draw a bootstrap sample Z^* of size N from the training data.
   - Grow a random-forest tree T_b to the bootstrapped data, by recursively repeating the following steps for each terminal node of the tree, until the minimum node size is reached.
     i. Select m variables at random from the p variables.
     ii. Pick the best variable/split-point among the m.
     iii. Split the node into two daughter nodes.
2. Output the ensemble of trees $\{T_b\}_1^B$

To make a prediction at a new point x:

Regression: $\hat{f}_{rf}^B(x) = \frac{1}{B}\sum_{b=1}^{B} T_b(x)$

Classification: $\widehat{C_b}(x)$ is the class prediction of the bth random-forest tree. $\hat{C}_{rf}^B(x) = majority\ vote\ \{\widehat{C_b}(x)\}_1^B$

11 Gradient Tree Boosting Model

Gradient tree boosting model combines multiple decision trees into a stronger model by repeatedly reweighting training examples to focus on the most problematic (Friedman, 2001; Hastie et al., 2009). A constant γ_j is assigned to each such region and the predictive rule is $x \in R_j \Rightarrow f(x) = \gamma_j$. Thus a tree can be formally expressed as

$$T(x, \Theta) = \sum_{j=1}^{J} \gamma_j I(x \in R_j)$$

with parameters $\Theta = \{R_j, \gamma_j\}_1^J$. J is usually treated as a meta-parameter. There are main steps in gradient tree boosting model as follow(Hastie et al., 2009):

1. Initialize $f_0(x) = argmin_\gamma \sum_{i=1}^{N} L(y_i, \gamma)$
2. For m = 1 to M:
   I. For i = 1, 2, ..., N compute
   - $r_{im} = -[\frac{\partial L(y_i, f(x_i))}{\partial f(x_i)}]_{f = f_{m-1}}$

   II. Fit a regression tree to the targets r_{im} giving terminal regions R_{jm}, the sizes of each of the constituent trees J_m, j = 1, 2, ..., J_m.

   III. For j = 1, 2, ..., J_m compute
   - $\gamma_{jm} = arg \min_{\gamma} \sum_{x_j \in R_{jm}} L(y_i, f_{m-1}(x_i) + \gamma)$

   IV. Update $f_m(x) = f_{m-1}(x) + \sum_{j=1}^{J_m} \gamma_{jm} I(x \in R_{jm})$
3. Output $\hat{f}(x) = f_M(x)$

12 Gaussian Processes

Multivariate normal distribution is as follow:

$$N(x|\mu,\Sigma) = \frac{1}{(2\pi)^{D/2}|\Sigma|^{1/2}} \exp\left(-\frac{1}{2}(x-\mu)^T\Sigma^{-1}(x-\mu)\right)$$

where D is the number of dimensions, x represents the variable, $\mu = \mathrm{E}[x]$ is the mean vector, and $\Sigma = \mathrm{cov}[\mathrm{x}]$ is the covariance matrix. Gaussian kernel function that is defined as

$$cov(x_i, x_j) = \exp\left(-\frac{(x_i - x_j)^2}{2}\right)$$

The Gaussian process model is a distribution over functions whose shape is defined by **K**. The standard Gaussian process model is as follow:

$$P(\boldsymbol{f}|\boldsymbol{X}) = N(\boldsymbol{f}|\boldsymbol{\mu},\boldsymbol{K})$$

Where the observed data points $\boldsymbol{X} = [x_1, \dots, x_n]$, $\boldsymbol{f} = [f(x_1), \dots, f(x_n)]$, the mean function $\boldsymbol{\mu} = [m(x_1), \dots, m(x_n)]$ and positive definite kernel function $K_{ij} = k(x_i, x_j)$

13 Reference

Efron, B., Hastie, T., Johnstone, I., Tibshirani, R., 2004. Least angle regression. Ann. Stat. 32. https://doi.org/10.1214/009053604000000067

Friedman, J.H., 2001. Greedy function approximation: A gradient boosting machine. Ann. Stat. 29. https://doi.org/10.1214/aos/1013203451

Hastie, T., Tibshirani, R., Friedman, J.H., 2009. The elements of statistical learning: data mining, inference, and prediction, 2nd ed. ed, Springer series in statistics. Springer, New York, NY.

Shalev-Shwartz, S., Tewari, A., 2011. Stochastic Methods for l_1-regularized Loss Minimization. J. Mach. Learn. Res. 12, 1865–1892.

Thung, K.-H., Wee, C.-Y., 2018. A brief review on multi-task learning. Multimed. Tools Appl. 77, 29705–29725. https://doi.org/10.1007/s11042-018-6463-x

Tibshirani, R., 1996. Regression Shrinkage and Selection Via the Lasso. J. R. Stat. Soc. Ser. B Methodol. 58, 267–288. https://doi.org/10.1111/j.2517-6161.1996.tb02080.x

Vladimir Vovk, 2013. Empirical inference. Springer Berlin Heidelberg, New York, NY.

Zhang, Y., Yang, Q., 2022. A Survey on Multi-Task Learning. IEEE Trans. Knowl. Data Eng. 34, 5586–5609. https://doi.org/10.1109/TKDE.2021.3070203

Zou, H., Hastie, T., 2005. Regularization and variable selection via the elastic net. J. R. Stat. Soc. Ser. B Stat. Methodol. 67, 301–320. https://doi.org/10.1111/j.1467-9868.2005.00503.x

Table S1 Computational time (CPU time) of the ML models to predict age. FVS: forward variable selection algorithm; MSE: mean squared error. Entries are sorted in order of ascending MSE values.

Model	Without FVS	With Boruta	With FVS
LassoLar	20.3 seconds	23.3 minutes	45.2 minutes
Random Forest	242.0 seconds	90.0 minutes	138.0 minutes
Gaussian Process	80.2 seconds	38.6 minutes	84.5 minutes
Ridge	15.6 seconds	16.7 minutes	30.7 minutes
Elastic net	48.7 seconds	26.3 minutes	47.2 minutes
Lars	18.2 seconds	19.6 minutes	32.1 minutes
Lasso	16.1 seconds	17.5 minutes	31.6 minutes
Kernel Ridge	68.6 seconds	25.2 minutes	72.8 minutes
Multitask Lasso	53.7 seconds	28.2 minutes	48.3 minutes
Decision Tree	81.2 seconds	39.7 minutes	90.7 minutes
Stochastic Gradient Descent	85.6 seconds	40.7 minutes	102.3 minutes

Table S2 Computational time (CPU time) of the ML models used to classify the male and female groups. FVS: forward variable selection algorithm. Entries are sorted in order of descending accuracy values**.**

Model	Without FVS	With Boruta	With FVS
Random Forest	150.0 seconds	90.5 minutes	132.0 minutes
Extreme Gradient Boosting	270.0 seconds	204.0 minutes	282.0 minutes
Logistic Regression with the Absolute Norm L1	19.0 seconds	20.6 minutes	33.6 minutes
Gradient Boosting	336.0 seconds	282.0 minutes	354.0 minutes
Extremely Randomized Trees	90.0 seconds	35.7 minutes	72.0 minutes
Decision Tree	84.0 seconds	33.4 minutes	66.0 minutes
Naive Bayes	21.0 seconds	22.4 minutes	34.8 minutes

Table S3 The number of commonly selected ROIs by the FVS and Boruta algorithms (overlaps between Tables S4 and S5) and their atlas names for each regression model. FVS: forward variable selection algorithm. Entries are sorted in ascending order of mean squared error (MSE) values (the best to worst from top to bottom).

Model	Number of ROIs	Name of ROIs
LassoLar	20	BNA006SFG_R_7_3, BNA079STG_L_6_6, BNA186CG_R_7_6, BNA225Str_L_6_4, BNA060PrG_R_6_4, BNA005SFG_L_7_3, BNA179CG_L_7_3, BNA083MTG_L_4_2, BNA011SFG_L_7_6, BNA216Hipp_R_2_1, BNA211Amyg_L_2_1, BNA110PhG_R_6_1, BNA116PhG_R_6_4, BNA191Cun_L_5_2, BNA218Hipp_R_2_2, BNA071STG_L_6_2, BNA232Tha_R_8_1, BNA235Tha_L_8_3, BNA234Tha_R_8_2, BNA233Tha_L_8_2,
Random Forest	30	BNA088MTG_R_4_4, BNA028MFG_R_7_7, BNA048OrG_R_6_4, BNA045OrG_L_6_3, BNA014SFG_R_7_7, BNA003SFG_L_7_2, BNA159PoG_L_4_3, BNA226Str_R_6_4, BNA013SFG_L_7_7, BNA012SFG_R_7_6, BNA055PrG_L_6_2, BNA138IPL_R_6_2, BNA087MTG_L_4_4, BNA051OrG_L_6_6, BNA139IPL_L_6_3, BNA127SPL_L_5_2, BNA122pSTS_R_2_1, BNA101ITG_L_7_7, BNA073STG_L_6_3, BNA067PCL_L_2_2, BNA050OrG_R_6_5, BNA124pSTS_R_2_2, BNA097ITG_L_7_5, BNA140IPL_R_6_3, BNA093ITG_L_7_3, BNA119PhG_L_6_6, BNA062PrG_R_6_5, BNA202OcG_R_4_2, BNA120PhG_R_6_6, BNA112PhG_R_6_2,
Gaussian Process	26	BNA157PoG_L_4_2, BNA203OcG_L_4_3, BNA200OcG_R_4_1, BNA198Cun_R_5_5, BNA206OcG_R_4_4, BNA094ITG_R_7_3, BNA034IFG_R_6_3, BNA103FuG_L_3_1, BNA170INS_R_6_4, BNA152PCun_R_4_3, BNA105FuG_L_3_2, BNA069STG_L_6_1, BNA076STG_R_6_4, BNA204OcG_R_4_3, BNA146IPL_R_6_6, BNA164INS_R_6_1, BNA109PhG_L_6_1, BNA072STG_R_6_2, BNA212Amyg_R_2_1, BNA117PhG_L_6_5, BNA239Tha_L_8_5, BNA217Hipp_L_2_2, BNA242Tha_R_8_6, BNA115PhG_L_6_4, BNA194Cun_R_5_3, BNA192Cun_R_5_2,
Ridge	33	BNA076STG_R_6_4, BNA227Str_L_6_5, BNA201OcG_L_4_2, BNA220Str_R_6_1, BNA228Str_R_6_5, BNA123pSTS_L_2_2, BNA197Cun_L_5_5, BNA190Cun_R_5_1, BNA068PCL_R_2_2, BNA058PrG_R_6_3, BNA155PoG_L_4_1, BNA144IPL_R_6_5, BNA086MTG_R_4_3, BNA107FuG_L_3_3, BNA075STG_L_6_4, BNA148PCun_R_4_1, BNA173INS_L_6_6, BNA223Str_L_6_3, BNA209sOcG_L_2_2, BNA189Cun_L_5_1, BNA036IFG_R_6_4, BNA061PrG_L_6_5, BNA125SPL_L_5_1, BNA172INS_R_6_5, BNA030IFG_R_6_1, BNA137IPL_L_6_2, BNA134SPL_R_5_5, BNA213Amyg_L_2_2, BNA240Tha_R_8_5, BNA118PhG_R_6_5, BNA241Tha_L_8_6, BNA214Amyg_R_2_2, BNA216Hipp_R_2_1,

Elastic net	10	BNA044OrG_R_6_2, BNA009SFG_L_7_5, BNA001SFG_L_7_1, BNA080STG_R_6_6, BNA025MFG_L_7_6, BNA027MFG_L_7_7, BNA088MTG_R_4_4, BNA028MFG_R_7_7, BNA048OrG_R_6_4, BNA045OrG_L_6_3,
Lars	6	BNA100ITG_R_7_6, BNA016MFG_R_7_1, BNA130SPL_R_5_3, BNA168INS_R_6_3, BNA196Cun_R_5_4, BNA037IFG_L_6_5,
Lasso	29	BNA199OcG_L_4_1, BNA167INS_L_6_3, BNA077STG_L_6_5, BNA085MTG_L_4_3, BNA127SPL_L_5_2, BNA122pSTS_R_2_1, BNA101ITG_L_7_7, BNA073STG_L_6_3, BNA067PCL_L_2_2, BNA050OrG_R_6_5, BNA124pSTS_R_2_2, BNA097ITG_L_7_5, BNA125SPL_L_5_1, BNA172INS_R_6_5, BNA030IFG_R_6_1, BNA137IPL_L_6_2, BNA134SPL_R_5_5, BNA213Amyg_L_2_2, BNA240Tha_R_8_5, BNA118PhG_R_6_5, BNA241Tha_L_8_6, BNA214Amyg_R_2_2, BNA216Hipp_R_2_1, BNA211Amyg_L_2_1, BNA110PhG_R_6_1, BNA116PhG_R_6_4, BNA191Cun_L_5_2, BNA218Hipp_R_2_2, BNA071STG_L_6_2,
Kernel Ridge	41	BNA048OrG_R_6_4, BNA045OrG_L_6_3, BNA014SFG_R_7_7, BNA003SFG_L_7_2, BNA159PoG_L_4_3, BNA226Str_R_6_4, BNA013SFG_L_7_7, BNA012SFG_R_7_6, BNA055PrG_L_6_2, BNA138IPL_R_6_2, BNA087MTG_L_4_4, BNA051OrG_L_6_6, BNA139IPL_L_6_3, BNA180CG_R_7_3, BNA017MFG_L_7_2, BNA175CG_L_7_1, BNA108FuG_R_3_3, BNA171INS_L_6_5, BNA174INS_R_6_6, BNA208sOcG_R_2_1, BNA142IPL_R_6_4, BNA144IPL_R_6_5, BNA086MTG_R_4_3, BNA107FuG_L_3_3, BNA075STG_L_6_4, BNA148PCun_R_4_1, BNA173INS_L_6_6, BNA223Str_L_6_3, BNA062PrG_R_6_5, BNA202OcG_R_4_2, BNA120PhG_R_6_6, BNA112PhG_R_6_2, BNA091ITG_L_7_2, BNA196Cun_R_5_4, BNA037IFG_L_6_5, BNA070STG_R_6_1, BNA098ITG_R_7_5, BNA151PCun_L_4_3, BNA090ITG_R_7_1, BNA104FuG_R_3_1, BNA177CG_L_7_2,
Multitask Lasso	14	BNA192Cun_R_5_2, BNA243Tha_L_8_7, BNA231Tha_L_8_1, BNA132SPL_R_5_4, BNA021MFG_L_7_4, BNA171INS_L_6_5, BNA174INS_R_6_6, BNA208sOcG_R_2_1, BNA024MFG_R_7_5, BNA007SFG_L_7_4, BNA150PCun_R_4_2, BNA036IFG_R_6_4, BNA061PrG_L_6_5, BNA125SPL_L_5_1,
Decision Tree	23	BNA209sOcG_L_2_2, BNA189Cun_L_5_1, BNA031IFG_L_6_2, BNA178CG_R_7_2, BNA224Str_R_6_3, BNA131SPL_L_5_4, BNA210sOcG_R_2_2, BNA121pSTS_L_2_1, BNA143IPL_L_6_5, BNA129SPL_L_5_3, BNA049OrG_L_6_5, BNA106FuG_R_3_2, BNA141IPL_L_6_4, BNA149PCun_L_4_2, BNA140IPL_R_6_3, BNA093ITG_L_7_3, BNA119PhG_L_6_6, BNA062PrG_R_6_5, BNA202OcG_R_4_2, BNA120PhG_R_6_6, BNA112PhG_R_6_2, BNA091ITG_L_7_2, BNA196Cun_R_5_4,
Stochastic Gradient Descent	36	BNA091ITG_L_7_2, BNA196Cun_R_5_4, BNA037IFG_L_6_5, BNA070STG_R_6_1, BNA098ITG_R_7_5, BNA151PCun_L_4_3, BNA090ITG_R_7_1, BNA104FuG_R_3_1, BNA113PhG_L_6_3,

		BNA114PhG_R_6_3, BNA204OcG_R_4_3, BNA207sOcG_L_2_1, BNA012SFG_R_7_6, BNA055PrG_L_6_2, BNA138IPL_R_6_2, BNA087MTG_L_4_4, BNA051OrG_L_6_6, BNA139IPL_L_6_3, BNA187CG_L_7_7, BNA024MFG_R_7_5, BNA007SFG_L_7_4, BNA150PCun_R_4_2, BNA036IFG_R_6_4, BNA061PrG_L_6_5, BNA125SPL_L_5_1, BNA172INS_R_6_5, BNA030IFG_R_6_1, BNA137IPL_L_6_2, BNA134SPL_R_5_5, BNA213Amyg_L_2_2, BNA240Tha_R_8_5, BNA118PhG_R_6_5, BNA241Tha_L_8_6, BNA214Amyg_R_2_2, BNA216Hipp_R_2_1, BNA211Amyg_L_2_1,

Table S4 The number of selected ROIs solely by the FVS algorithm (including the ROIs in Table S3) and their atlas names for each regression model. FVS: forward variable selection algorithm. Entries are sorted in ascending order of mean squared error (MSE) values (the best to worst from top to bottom).

Model	Number of ROIs	Name of ROIs
LassoLar	54	BNA046OrG_R_6_3, BNA043OrG_L_6_2, BNA230Str_R_6_6, BNA010SFG_R_7_5, BNA188CG_R_7_7, BNA006SFG_R_7_3, BNA079STG_L_6_6, BNA082MTG_R_4_1, BNA186CG_R_7_6, BNA026MFG_R_7_6, BNA225Str_L_6_4, BNA060PrG_R_6_4, BNA005SFG_L_7_3, BNA179CG_L_7_3, BNA083MTG_L_4_2, BNA011SFG_L_7_6, BNA092ITG_R_7_2, BNA161PoG_L_4_4, BNA015MFG_L_7_1, BNA063PrG_L_6_6, BNA056PrG_R_6_2, BNA135IPL_L_6_1, BNA095ITG_L_7_4, BNA035IFG_L_6_4, BNA229Str_L_6_6, BNA221Str_L_6_2, BNA059PrG_L_6_4, BNA187CG_L_7_7, BNA024MFG_R_7_5, BNA007SFG_L_7_4, BNA150PCun_R_4_2, BNA036IFG_R_6_4, BNA061PrG_L_6_5, BNA125SPL_L_5_1, BNA172INS_R_6_5, BNA030IFG_R_6_1, BNA137IPL_L_6_2, BNA134SPL_R_5_5, BNA213Amyg_L_2_2, BNA240Tha_R_8_5, BNA118PhG_R_6_5, BNA241Tha_L_8_6, BNA214Amyg_R_2_2, BNA216Hipp_R_2_1, BNA211Amyg_L_2_1, BNA110PhG_R_6_1, BNA116PhG_R_6_4, BNA191Cun_L_5_2, BNA218Hipp_R_2_2, BNA071STG_L_6_2, BNA232Tha_R_8_1, BNA235Tha_L_8_3, BNA234Tha_R_8_2, BNA233Tha_L_8_2,
Random Forest	63	BNA044OrG_R_6_2, BNA009SFG_L_7_5, BNA001SFG_L_7_1, BNA080STG_R_6_6, BNA025MFG_L_7_6, BNA027MFG_L_7_7, BNA088MTG_R_4_4, BNA028MFG_R_7_7, BNA048OrG_R_6_4, BNA045OrG_L_6_3, BNA014SFG_R_7_7, BNA003SFG_L_7_2, BNA159PoG_L_4_3, BNA226Str_R_6_4, BNA013SFG_L_7_7, BNA012SFG_R_7_6, BNA055PrG_L_6_2, BNA138IPL_R_6_2, BNA087MTG_L_4_4, BNA051OrG_L_6_6, BNA139IPL_L_6_3, BNA180CG_R_7_3, BNA017MFG_L_7_2, BNA175CG_L_7_1, BNA108FuG_R_3_3, BNA019MFG_L_7_3, BNA047OrG_L_6_4, BNA023MFG_L_7_5, BNA002SFG_R_7_1, BNA042OrG_R_6_1, BNA004SFG_R_7_2, BNA074STG_R_6_3, BNA127SPL_L_5_2, BNA122pSTS_R_2_1, BNA101ITG_L_7_7, BNA073STG_L_6_3, BNA067PCL_L_2_2, BNA050OrG_R_6_5, BNA124pSTS_R_2_2, BNA097ITG_L_7_5, BNA140IPL_R_6_3, BNA093ITG_L_7_3, BNA119PhG_L_6_6, BNA062PrG_R_6_5, BNA202OcG_R_4_2, BNA120PhG_R_6_6, BNA112PhG_R_6_2, BNA091ITG_L_7_2, BNA196Cun_R_5_4, BNA118PhG_R_6_5, BNA241Tha_L_8_6, BNA214Amyg_R_2_2, BNA216Hipp_R_2_1, BNA211Amyg_L_2_1, BNA110PhG_R_6_1, BNA116PhG_R_6_4, BNA191Cun_L_5_2,

		BNA218Hipp_R_2_2, BNA071STG_L_6_2, BNA232Tha_R_8_1, BNA235Tha_L_8_3, BNA234Tha_R_8_2, BNA233Tha_L_8_2,
Gaussian Process	78	BNA057PrG_L_6_3, BNA040IFG_R_6_6, BNA111PhG_L_6_2, BNA157PoG_L_4_2, BNA203OcG_L_4_3, BNA200OcG_R_4_1, BNA198Cun_R_5_5, BNA206OcG_R_4_4, BNA094ITG_R_7_3, BNA034IFG_R_6_3, BNA103FuG_L_3_1, BNA170INS_R_6_4, BNA152PCun_R_4_3, BNA105FuG_L_3_2, BNA069STG_L_6_1, BNA076STG_R_6_4, BNA227Str_L_6_5, BNA201OcG_L_4_2, BNA220Str_R_6_1, BNA228Str_R_6_5, BNA123pSTS_L_2_2, BNA197Cun_L_5_5, BNA190Cun_R_5_1, BNA113PhG_L_6_3, BNA114PhG_R_6_3, BNA204OcG_R_4_3, BNA146IPL_R_6_6, BNA164INS_R_6_1, BNA109PhG_L_6_1, BNA072STG_R_6_2, BNA212Amyg_R_2_1, BNA117PhG_L_6_5, BNA239Tha_L_8_5, BNA217Hipp_L_2_2, BNA242Tha_R_8_6, BNA115PhG_L_6_4, BNA194Cun_R_5_3, BNA192Cun_R_5_2, BNA243Tha_L_8_7, BNA231Tha_L_8_1, BNA246Tha_R_8_8, BNA245Tha_L_8_8, BNA244Tha_R_8_7, BNA237Tha_L_8_4, BNA236Tha_R_8_3, BNA238Tha_R_8_4, BNA046OrG_R_6_3, BNA043OrG_L_6_2, BNA230Str_R_6_6, BNA010SFG_R_7_5, BNA188CG_R_7_7, BNA006SFG_R_7_3, BNA079STG_L_6_6, BNA082MTG_R_4_1, BNA186CG_R_7_6, BNA026MFG_R_7_6, BNA225Str_L_6_4, BNA060PrG_R_6_4, BNA005SFG_L_7_3, BNA179CG_L_7_3, BNA083MTG_L_4_2, BNA229Str_L_6_6, BNA221Str_L_6_2, BNA059PrG_L_6_4, BNA187CG_L_7_7, BNA024MFG_R_7_5, BNA007SFG_L_7_4, BNA150PCun_R_4_2, BNA036IFG_R_6_4, BNA061PrG_L_6_5, BNA125SPL_L_5_1, BNA172INS_R_6_5, BNA030IFG_R_6_1, BNA204OcG_R_4_3, BNA207sOcG_L_2_1, BNA089ITG_L_7_1, BNA219Str_L_6_1, BNA065PCL_L_2_1,
Ridge	81	BNA033IFG_L_6_3, BNA066PCL_R_2_1, BNA205OcG_L_4_4, BNA128SPL_R_5_2, BNA169INS_L_6_4, BNA099ITG_L_7_6, BNA057PrG_L_6_3, BNA040IFG_R_6_6, BNA111PhG_L_6_2, BNA157PoG_L_4_2, BNA203OcG_L_4_3, BNA200OcG_R_4_1, BNA198Cun_R_5_5, BNA206OcG_R_4_4, BNA094ITG_R_7_3, BNA034IFG_R_6_3, BNA103FuG_L_3_1, BNA170INS_R_6_4, BNA152PCun_R_4_3, BNA105FuG_L_3_2, BNA069STG_L_6_1, BNA076STG_R_6_4, BNA227Str_L_6_5, BNA201OcG_L_4_2, BNA220Str_R_6_1, BNA228Str_R_6_5, BNA123pSTS_L_2_2, BNA197Cun_L_5_5, BNA190Cun_R_5_1, BNA113PhG_L_6_3, BNA114PhG_R_6_3, BNA204OcG_R_4_3, BNA136IPL_R_6_1, BNA084MTG_R_4_2, BNA064PrG_R_6_6, BNA096ITG_R_7_4, BNA153PCun_L_4_4, BNA053PrG_L_6_1, BNA147PCun_L_4_1, BNA068PCL_R_2_2, BNA058PrG_R_6_3, BNA155PoG_L_4_1, BNA144IPL_R_6_5, BNA086MTG_R_4_3, BNA107FuG_L_3_3, BNA075STG_L_6_4, BNA148PCun_R_4_1, BNA173INS_L_6_6, BNA223Str_L_6_3, BNA209sOcG_L_2_2, BNA189Cun_L_5_1, BNA036IFG_R_6_4, BNA061PrG_L_6_5, BNA125SPL_L_5_1,

		BNA172INS_R_6_5, BNA030IFG_R_6_1, BNA137IPL_L_6_2, BNA134SPL_R_5_5, BNA213Amyg_L_2_2, BNA240Tha_R_8_5, BNA118PhG_R_6_5, BNA241Tha_L_8_6, BNA214Amyg_R_2_2, BNA216Hipp_R_2_1, BNA211Amyg_L_2_1, BNA110PhG_R_6_1, BNA116PhG_R_6_4, BNA191Cun_L_5_2, BNA218Hipp_R_2_2, BNA071STG_L_6_2, BNA232Tha_R_8_1, BNA235Tha_L_8_3, BNA234Tha_R_8_2, BNA233Tha_L_8_2, BNA026MFG_R_7_6, BNA225Str_L_6_4, BNA060PrG_R_6_4, BNA005SFG_L_7_3, BNA068PCL_R_2_2, BNA058PrG_R_6_3, BNA155PoG_L_4_1,
Elastic net	73	BNA167INS_L_6_3, BNA077STG_L_6_5, BNA085MTG_L_4_3, BNA127SPL_L_5_2, BNA122pSTS_R_2_1, BNA101ITG_L_7_7, BNA073STG_L_6_3, BNA067PCL_L_2_2, BNA050OrG_R_6_5, BNA124pSTS_R_2_2, BNA097ITG_L_7_5, BNA140IPL_R_6_3, BNA093ITG_L_7_3, BNA105FuG_L_3_2, BNA069STG_L_6_1, BNA076STG_R_6_4, BNA227Str_L_6_5, BNA201OcG_L_4_2, BNA220Str_R_6_1, BNA228Str_R_6_5, BNA123pSTS_L_2_2, BNA197Cun_L_5_5, BNA190Cun_R_5_1, BNA113PhG_L_6_3, BNA114PhG_R_6_3, BNA204OcG_R_4_3, BNA207sOcG_L_2_1, BNA089ITG_L_7_1, BNA044OrG_R_6_2, BNA009SFG_L_7_5, BNA001SFG_L_7_1, BNA080STG_R_6_6, BNA025MFG_L_7_6, BNA027MFG_L_7_7, BNA088MTG_R_4_4, BNA028MFG_R_7_7, BNA048OrG_R_6_4, BNA045OrG_L_6_3, BNA030IFG_R_6_1, BNA137IPL_L_6_2, BNA134SPL_R_5_5, BNA213Amyg_L_2_2, BNA240Tha_R_8_5, BNA118PhG_R_6_5, BNA241Tha_L_8_6, BNA214Amyg_R_2_2, BNA216Hipp_R_2_1, BNA211Amyg_L_2_1, BNA110PhG_R_6_1, BNA116PhG_R_6_4, BNA191Cun_L_5_2, BNA218Hipp_R_2_2, BNA071STG_L_6_2, BNA232Tha_R_8_1, BNA235Tha_L_8_3, BNA234Tha_R_8_2, BNA233Tha_L_8_2, BNA212Amyg_R_2_1, BNA117PhG_L_6_5, BNA239Tha_L_8_5, BNA217Hipp_L_2_2, BNA242Tha_R_8_6, BNA115PhG_L_6_4, BNA194Cun_R_5_3, BNA192Cun_R_5_2, BNA243Tha_L_8_7, BNA231Tha_L_8_1, BNA246Tha_R_8_8, BNA245Tha_L_8_8, BNA244Tha_R_8_7, BNA237Tha_L_8_4, BNA236Tha_R_8_3, BNA238Tha_R_8_4,
Lars	68	BNA173INS_L_6_6, BNA223Str_L_6_3, BNA209sOcG_L_2_2, BNA189Cun_L_5_1, BNA031IFG_L_6_2, BNA178CG_R_7_2, BNA224Str_R_6_3, BNA131SPL_L_5_4, BNA210sOcG_R_2_2, BNA121pSTS_L_2_1, BNA143IPL_L_6_5, BNA129SPL_L_5_3, BNA049OrG_L_6_5, BNA100ITG_R_7_6, BNA016MFG_R_7_1, BNA130SPL_R_5_3, BNA168INS_R_6_3, BNA081MTG_L_4_1, BNA041OrG_L_6_1, BNA160PoG_R_4_3, BNA222Str_R_6_2, BNA038IFG_R_6_5, BNA102ITG_R_7_7, BNA054PrG_R_6_1, BNA154PCun_R_4_4, BNA051OrG_L_6_6, BNA139IPL_L_6_3, BNA180CG_R_7_3, BNA017MFG_L_7_2, BNA175CG_L_7_1, BNA108FuG_R_3_3, BNA019MFG_L_7_3, BNA047OrG_L_6_4, BNA023MFG_L_7_5, BNA002SFG_R_7_1, BNA140IPL_R_6_3,

		BNA093ITG_L_7_3, BNA119PhG_L_6_6, BNA062PrG_R_6_5, BNA202OcG_R_4_2, BNA120PhG_R_6_6, BNA112PhG_R_6_2, BNA091ITG_L_7_2, BNA196Cun_R_5_4, BNA037IFG_L_6_5, BNA046OrG_R_6_3, BNA043OrG_L_6_2, BNA230Str_R_6_6, BNA010SFG_R_7_5, BNA188CG_R_7_7, BNA006SFG_R_7_3, BNA079STG_L_6_6, BNA082MTG_R_4_1, BNA186CG_R_7_6, BNA026MFG_R_7_6, BNA225Str_L_6_4, BNA060PrG_R_6_4, BNA005SFG_L_7_3, BNA179CG_L_7_3, BNA083MTG_L_4_2, BNA198Cun_R_5_5, BNA206OcG_R_4_4, BNA094ITG_R_7_3, BNA034IFG_R_6_3, BNA103FuG_L_3_1, BNA170INS_R_6_4, BNA152PCun_R_4_3, BNA105FuG_L_3_2
Lasso	59	BNA109PhG_L_6_1, BNA072STG_R_6_2, BNA212Amyg_R_2_1, BNA117PhG_L_6_5, BNA239Tha_L_8_5, BNA217Hipp_L_2_2, BNA242Tha_R_8_6, BNA115PhG_L_6_4, BNA194Cun_R_5_3, BNA192Cun_R_5_2, BNA243Tha_L_8_7, BNA231Tha_L_8_1, BNA246Tha_R_8_8, BNA245Tha_L_8_8, BNA040IFG_R_6_6, BNA111PhG_L_6_2, BNA157PoG_L_4_2, BNA203OcG_L_4_3, BNA200OcG_R_4_1, BNA198Cun_R_5_5, BNA206OcG_R_4_4, BNA094ITG_R_7_3, BNA034IFG_R_6_3, BNA103FuG_L_3_1, BNA170INS_R_6_4, BNA152PCun_R_4_3, BNA105FuG_L_3_2, BNA069STG_L_6_1, BNA076STG_R_6_4, BNA227Str_L_6_5, BNA199OcG_L_4_1, BNA167INS_L_6_3, BNA077STG_L_6_5, BNA085MTG_L_4_3, BNA127SPL_L_5_2, BNA122pSTS_R_2_1, BNA101ITG_L_7_7, BNA073STG_L_6_3, BNA067PCL_L_2_2, BNA050OrG_R_6_5, BNA124pSTS_R_2_2, BNA097ITG_L_7_5, BNA125SPL_L_5_1, BNA172INS_R_6_5, BNA030IFG_R_6_1, BNA137IPL_L_6_2, BNA134SPL_R_5_5, BNA213Amyg_L_2_2, BNA240Tha_R_8_5, BNA118PhG_R_6_5, BNA241Tha_L_8_6, BNA214Amyg_R_2_2, BNA216Hipp_R_2_1, BNA211Amyg_L_2_1, BNA110PhG_R_6_1, BNA116PhG_R_6_4, BNA191Cun_L_5_2, BNA218Hipp_R_2_2, BNA071STG_L_6_2,
Kernel Ridge	71	BNA027MFG_L_7_7, BNA088MTG_R_4_4, BNA028MFG_R_7_7, BNA048OrG_R_6_4, BNA045OrG_L_6_3, BNA014SFG_R_7_7, BNA003SFG_L_7_2, BNA159PoG_L_4_3, BNA226Str_R_6_4, BNA013SFG_L_7_7, BNA012SFG_R_7_6, BNA055PrG_L_6_2, BNA138IPL_R_6_2, BNA087MTG_L_4_4, BNA051OrG_L_6_6, BNA139IPL_L_6_3, BNA180CG_R_7_3, BNA017MFG_L_7_2, BNA175CG_L_7_1, BNA108FuG_R_3_3, BNA155PoG_L_4_1, BNA029IFG_L_6_1, BNA132SPL_R_5_4, BNA021MFG_L_7_4, BNA171INS_L_6_5, BNA174INS_R_6_6, BNA208sOcG_R_2_1, BNA142IPL_R_6_4, BNA144IPL_R_6_5, BNA086MTG_R_4_3, BNA107FuG_L_3_3, BNA075STG_L_6_4, BNA148PCun_R_4_1, BNA173INS_L_6_6, BNA223Str_L_6_3, BNA209sOcG_L_2_2, BNA119PhG_L_6_6, BNA062PrG_R_6_5, BNA202OcG_R_4_2, BNA120PhG_R_6_6, BNA112PhG_R_6_2, BNA091ITG_L_7_2, BNA196Cun_R_5_4, BNA037IFG_L_6_5, BNA070STG_R_6_1,

		BNA098ITG_R_7_5, BNA151PCun_L_4_3, BNA090ITG_R_7_1, BNA104FuG_R_3_1, BNA177CG_L_7_2, BNA158PoG_R_4_2, BNA033IFG_L_6_3, BNA066PCL_R_2_1, BNA205OcG_L_4_4, BNA010SFG_R_7_5, BNA188CG_R_7_7, BNA006SFG_R_7_3, BNA079STG_L_6_6, BNA082MTG_R_4_1, BNA186CG_R_7_6, BNA026MFG_R_7_6, BNA225Str_L_6_4, BNA060PrG_R_6_4, BNA005SFG_L_7_3, BNA179CG_L_7_3, BNA083MTG_L_4_2, BNA011SFG_L_7_6, BNA092ITG_R_7_2, BNA161PoG_L_4_4, BNA015MFG_L_7_1, BNA063PrG_L_6_6,
Multitask Lasso	52	BNA212Amyg_R_2_1, BNA117PhG_L_6_5, BNA239Tha_L_8_5, BNA217Hipp_L_2_2, BNA242Tha_R_8_6, BNA115PhG_L_6_4, BNA194Cun_R_5_3, BNA192Cun_R_5_2, BNA243Tha_L_8_7, BNA231Tha_L_8_1, BNA246Tha_R_8_8, BNA245Tha_L_8_8, BNA244Tha_R_8_7, BNA237Tha_L_8_4, BNA236Tha_R_8_3, BNA238Tha_R_8_4, BNA033IFG_L_6_3, BNA066PCL_R_2_1, BNA205OcG_L_4_4, BNA128SPL_R_5_2, BNA169INS_L_6_4, BNA099ITG_L_7_6, BNA057PrG_L_6_3, BNA040IFG_R_6_6, BNA111PhG_L_6_2, BNA157PoG_L_4_2, BNA203OcG_L_4_3, BNA200OcG_R_4_1, BNA198Cun_R_5_5, BNA206OcG_R_4_4, BNA132SPL_R_5_4, BNA021MFG_L_7_4, BNA171INS_L_6_5, BNA174INS_R_6_6, BNA208sOcG_R_2_1, BNA142IPL_R_6_4, BNA144IPL_R_6_5, BNA086MTG_R_4_3, BNA107FuG_L_3_3, BNA075STG_L_6_4, BNA148PCun_R_4_1, BNA173INS_L_6_6, BNA223Str_L_6_3, BNA209sOcG_L_2_2, BNA059PrG_L_6_4, BNA187CG_L_7_7, BNA024MFG_R_7_5, BNA007SFG_L_7_4, BNA150PCun_R_4_2, BNA036IFG_R_6_4, BNA061PrG_L_6_5, BNA125SPL_L_5_1,
Decision Tree	76	BNA209sOcG_L_2_2, BNA189Cun_L_5_1, BNA031IFG_L_6_2, BNA178CG_R_7_2, BNA224Str_R_6_3, BNA131SPL_L_5_4, BNA210sOcG_R_2_2, BNA121pSTS_L_2_1, BNA143IPL_L_6_5, BNA129SPL_L_5_3, BNA049OrG_L_6_5, BNA106FuG_R_3_2, BNA141IPL_L_6_4, BNA149PCun_L_4_2, BNA126SPL_R_5_1, BNA133SPL_L_5_5, BNA195Cun_L_5_4, BNA032IFG_R_6_2, BNA140IPL_R_6_3, BNA093ITG_L_7_3, BNA119PhG_L_6_6, BNA062PrG_R_6_5, BNA202OcG_R_4_2, BNA120PhG_R_6_6, BNA112PhG_R_6_2, BNA091ITG_L_7_2, BNA196Cun_R_5_4, BNA037IFG_L_6_5, BNA070STG_R_6_1, BNA098ITG_R_7_5, BNA151PCun_L_4_3, BNA090ITG_R_7_1, BNA104FuG_R_3_1, BNA177CG_L_7_2, BNA158PoG_R_4_2, BNA033IFG_L_6_3, BNA044OrG_R_6_2, BNA009SFG_L_7_5, BNA001SFG_L_7_1, BNA080STG_R_6_6, BNA025MFG_L_7_6, BNA027MFG_L_7_7, BNA088MTG_R_4_4, BNA028MFG_R_7_7, BNA048OrG_R_6_4, BNA045OrG_L_6_3, BNA014SFG_R_7_7, BNA003SFG_L_7_2, BNA240Tha_R_8_5, BNA118PhG_R_6_5, BNA241Tha_L_8_6, BNA214Amyg_R_2_2, BNA216Hipp_R_2_1, BNA211Amyg_L_2_1, BNA110PhG_R_6_1, BNA116PhG_R_6_4, BNA191Cun_L_5_2,

		BNA218Hipp_R_2_2, BNA071STG_L_6_2, BNA232Tha_R_8_1, BNA235Tha_L_8_3, BNA234Tha_R_8_2, BNA233Tha_L_8_2, BNA230Str_R_6_6, BNA010SFG_R_7_5, BNA188CG_R_7_7, BNA006SFG_R_7_3, BNA226Str_R_6_4, BNA013SFG_L_7_7, BNA012SFG_R_7_6, BNA055PrG_L_6_2, BNA138IPL_R_6_2, BNA087MTG_L_4_4, BNA051OrG_L_6_6, BNA139IPL_L_6_3, BNA180CG_R_7_3,
Stochastic Gradient Descent	80	BNA140IPL_R_6_3, BNA093ITG_L_7_3, BNA119PhG_L_6_6, BNA062PrG_R_6_5, BNA202OcG_R_4_2, BNA120PhG_R_6_6, BNA112PhG_R_6_2, BNA091ITG_L_7_2, BNA196Cun_R_5_4, BNA037IFG_L_6_5, BNA070STG_R_6_1, BNA098ITG_R_7_5, BNA151PCun_L_4_3, BNA090ITG_R_7_1, BNA104FuG_R_3_1, BNA069STG_L_6_1, BNA076STG_R_6_4, BNA227Str_L_6_5, BNA201OcG_L_4_2, BNA220Str_R_6_1, BNA228Str_R_6_5, BNA123pSTS_L_2_2, BNA197Cun_L_5_5, BNA190Cun_R_5_1, BNA113PhG_L_6_3, BNA114PhG_R_6_3, BNA204OcG_R_4_3, BNA207sOcG_L_2_1, BNA089ITG_L_7_1, BNA219Str_L_6_1, BNA194Cun_R_5_3, BNA192Cun_R_5_2, BNA243Tha_L_8_7, BNA231Tha_L_8_1, BNA246Tha_R_8_8, BNA245Tha_L_8_8, BNA244Tha_R_8_7, BNA237Tha_L_8_4, BNA236Tha_R_8_3, BNA238Tha_R_8_4, BNA014SFG_R_7_7, BNA003SFG_L_7_2, BNA159PoG_L_4_3, BNA226Str_R_6_4, BNA013SFG_L_7_7, BNA012SFG_R_7_6, BNA055PrG_L_6_2, BNA138IPL_R_6_2, BNA087MTG_L_4_4, BNA051OrG_L_6_6, BNA139IPL_L_6_3, BNA187CG_L_7_7, BNA024MFG_R_7_5, BNA007SFG_L_7_4, BNA150PCun_R_4_2, BNA036IFG_R_6_4, BNA061PrG_L_6_5, BNA125SPL_L_5_1, BNA172INS_R_6_5, BNA030IFG_R_6_1, BNA137IPL_L_6_2, BNA134SPL_R_5_5, BNA213Amyg_L_2_2, BNA240Tha_R_8_5, BNA118PhG_R_6_5, BNA241Tha_L_8_6, BNA214Amyg_R_2_2, BNA216Hipp_R_2_1, BNA211Amyg_L_2_1, BNA110PhG_R_6_1, BNA116PhG_R_6_4, BNA191Cun_L_5_2, BNA218Hipp_R_2_2, BNA071STG_L_6_2, BNA232Tha_R_8_1, BNA235Tha_L_8_3, BNA234Tha_R_8_2, BNA233Tha_L_8_2, BNA113PhG_L_6_3, BNA114PhG_R_6_3,

Table S5 The number of ROIs solely selected by the Boruta algorithm (including the ROIs in Table S3) and their atlas names for each regression model. Entries are sorted in ascending order of mean squared error (MSE) values (the best to worst from top to bottom)

Model	Number of ROIs	Name of ROIs
LassoLar	62	BNA006SFG_R_7_3, BNA079STG_L_6_6, BNA082MTG_R_4_1, BNA186CG_R_7_6, BNA026MFG_R_7_6, BNA225Str_L_6_4, BNA060PrG_R_6_4, BNA005SFG_L_7_3, BNA179CG_L_7_3, BNA083MTG_L_4_2, BNA011SFG_L_7_6, BNA003SFG_L_7_2, BNA159PoG_L_4_3, BNA226Str_R_6_4, BNA013SFG_L_7_7, BNA012SFG_R_7_6, BNA055PrG_L_6_2, BNA138IPL_R_6_2, BNA087MTG_L_4_4, BNA051OrG_L_6_6, BNA139IPL_L_6_3, BNA180CG_R_7_3, BNA017MFG_L_7_2, BNA175CG_L_7_1, BNA108FuG_R_3_3, BNA019MFG_L_7_3, BNA047OrG_L_6_4, BNA023MFG_L_7_5, BNA002SFG_R_7_1, BNA216Hipp_R_2_1, BNA211Amyg_L_2_1, BNA110PhG_R_6_1, BNA116PhG_R_6_4, BNA191Cun_L_5_2, BNA218Hipp_R_2_2, BNA071STG_L_6_2, BNA232Tha_R_8_1, BNA235Tha_L_8_3, BNA234Tha_R_8_2, BNA233Tha_L_8_2, BNA039IFG_L_6_6, BNA156PoG_R_4_1, BNA078STG_R_6_5, BNA022MFG_R_7_4, BNA184CG_R_7_5, BNA020MFG_R_7_3, BNA182CG_R_7_4, BNA018MFG_R_7_2, BNA165INS_L_6_2, BNA176CG_R_7_1, BNA181CG_L_7_4, BNA100ITG_R_7_6, BNA016MFG_R_7_1, BNA130SPL_R_5_3, BNA168INS_R_6_3, BNA081MTG_L_4_1, BNA041OrG_L_6_1, BNA160PoG_R_4_3, BNA222Str_R_6_2, BNA038IFG_R_6_5, BNA102ITG_R_7_7,
Random Forest	75	BNA127SPL_L_5_2, BNA122pSTS_R_2_1, BNA101ITG_L_7_7, BNA073STG_L_6_3, BNA067PCL_L_2_2, BNA050OrG_R_6_5, BNA124pSTS_R_2_2, BNA097ITG_L_7_5, BNA140IPL_R_6_3, BNA093ITG_L_7_3, BNA119PhG_L_6_6, BNA062PrG_R_6_5, BNA202OcG_R_4_2, BNA120PhG_R_6_6, BNA112PhG_R_6_2, BNA090ITG_R_7_1, BNA104FuG_R_3_1, BNA177CG_L_7_2, BNA158PoG_R_4_2, BNA033IFG_L_6_3, BNA066PCL_R_2_1, BNA205OcG_L_4_4, BNA128SPL_R_5_2, BNA169INS_L_6_4, BNA099ITG_L_7_6, BNA057PrG_L_6_3, BNA088MTG_R_4_4, BNA028MFG_R_7_7, BNA048OrG_R_6_4, BNA045OrG_L_6_3, BNA014SFG_R_7_7, BNA003SFG_L_7_2, BNA159PoG_L_4_3, BNA226Str_R_6_4, BNA013SFG_L_7_7, BNA012SFG_R_7_6, BNA055PrG_L_6_2, BNA138IPL_R_6_2, BNA087MTG_L_4_4, BNA051OrG_L_6_6, BNA139IPL_L_6_3, BNA076STG_R_6_4, BNA227Str_L_6_5, BNA201OcG_L_4_2, BNA220Str_R_6_1, BNA228Str_R_6_5, BNA123pSTS_L_2_2, BNA197Cun_L_5_5, BNA190Cun_R_5_1, BNA113PhG_L_6_3, BNA114PhG_R_6_3, BNA204OcG_R_4_3, BNA207sOcG_L_2_1, BNA089ITG_L_7_1,

		BNA219Str_L_6_1, BNA065PCL_L_2_1, BNA145IPL_L_6_6, BNA193Cun_L_5_3, BNA215Hipp_L_2_1, BNA192Cun_R_5_2, BNA243Tha_L_8_7, BNA231Tha_L_8_1, BNA246Tha_R_8_8, BNA245Tha_L_8_8, BNA244Tha_R_8_7, BNA237Tha_L_8_4, BNA236Tha_R_8_3, BNA238Tha_R_8_4, BNA046OrG_R_6_3, BNA043OrG_L_6_2, BNA230Str_R_6_6, BNA010SFG_R_7_5, BNA188CG_R_7_7, BNA006SFG_R_7_3, BNA079STG_L_6_6,
Gaussian Process	83	BNA102ITG_R_7_7, BNA054PrG_R_6_1, BNA154PCun_R_4_4, BNA008SFG_R_7_4, BNA166INS_R_6_2, BNA153PCun_L_4_4, BNA053PrG_L_6_1, BNA147PCun_L_4_1, BNA068PCL_R_2_2, BNA058PrG_R_6_3, BNA155PoG_L_4_1, BNA029IFG_L_6_1, BNA132SPL_R_5_4, BNA021MFG_L_7_4, BNA171INS_L_6_5, BNA133SPL_L_5_5, BNA195Cun_L_5_4, BNA032IFG_R_6_2, BNA199OcG_L_4_1, BNA167INS_L_6_3, BNA077STG_L_6_5, BNA085MTG_L_4_3, BNA127SPL_L_5_2, BNA122pSTS_R_2_1, BNA101ITG_L_7_7, BNA073STG_L_6_3, BNA067PCL_L_2_2, BNA050OrG_R_6_5, BNA157PoG_L_4_2, BNA203OcG_L_4_3, BNA200OcG_R_4_1, BNA198Cun_R_5_5, BNA206OcG_R_4_4, BNA094ITG_R_7_3, BNA034IFG_R_6_3, BNA103FuG_L_3_1, BNA170INS_R_6_4, BNA152PCun_R_4_3, BNA105FuG_L_3_2, BNA069STG_L_6_1, BNA076STG_R_6_4, BNA215Hipp_L_2_1, BNA163INS_L_6_1, BNA146IPL_R_6_6, BNA164INS_R_6_1, BNA109PhG_L_6_1, BNA072STG_R_6_2, BNA212Amyg_R_2_1, BNA117PhG_L_6_5, BNA239Tha_L_8_5, BNA217Hipp_L_2_2, BNA242Tha_R_8_6, BNA115PhG_L_6_4, BNA194Cun_R_5_3, BNA192Cun_R_5_2, BNA079STG_L_6_6, BNA082MTG_R_4_1, BNA186CG_R_7_6, BNA026MFG_R_7_6, BNA225Str_L_6_4, BNA060PrG_R_6_4, BNA005SFG_L_7_3, BNA179CG_L_7_3, BNA083MTG_L_4_2, BNA011SFG_L_7_6, BNA092ITG_R_7_2, BNA161PoG_L_4_4, BNA015MFG_L_7_1, BNA063PrG_L_6_6, BNA056PrG_R_6_2, BNA135IPL_L_6_1, BNA095ITG_L_7_4, BNA035IFG_L_6_4, BNA229Str_L_6_6, BNA221Str_L_6_2, BNA059PrG_L_6_4, BNA231Tha_L_8_1, BNA246Tha_R_8_8, BNA245Tha_L_8_8, BNA244Tha_R_8_7, BNA237Tha_L_8_4, BNA236Tha_R_8_3, BNA238Tha_R_8_4,
Ridge	79	BNA013SFG_L_7_7, BNA012SFG_R_7_6, BNA055PrG_L_6_2, BNA138IPL_R_6_2, BNA087MTG_L_4_4, BNA051OrG_L_6_6, BNA139IPL_L_6_3, BNA180CG_R_7_3, BNA017MFG_L_7_2, BNA175CG_L_7_1, BNA108FuG_R_3_3, BNA019MFG_L_7_3, BNA047OrG_L_6_4, BNA023MFG_L_7_5, BNA039IFG_L_6_6, BNA156PoG_R_4_1, BNA078STG_R_6_5, BNA022MFG_R_7_4, BNA184CG_R_7_5, BNA020MFG_R_7_3, BNA182CG_R_7_4, BNA018MFG_R_7_2, BNA165INS_L_6_2, BNA176CG_R_7_1, BNA181CG_L_7_4, BNA100ITG_R_7_6, BNA016MFG_R_7_1, BNA171INS_L_6_5, BNA174INS_R_6_6, BNA208sOcG_R_2_1, BNA142IPL_R_6_4, BNA144IPL_R_6_5, BNA086MTG_R_4_3,

		BNA107FuG_L_3_3, BNA075STG_L_6_4, BNA148PCun_R_4_1, BNA173INS_L_6_6, BNA223Str_L_6_3, BNA209sOcG_L_2_2, BNA189Cun_L_5_1, BNA031IFG_L_6_2, BNA127SPL_L_5_2, BNA122pSTS_R_2_1, BNA101ITG_L_7_7, BNA073STG_L_6_3, BNA067PCL_L_2_2, BNA050OrG_R_6_5, BNA124pSTS_R_2_2, BNA097ITG_L_7_5, BNA140IPL_R_6_3, BNA093ITG_L_7_3, BNA119PhG_L_6_6, BNA062PrG_R_6_5, BNA202OcG_R_4_2, BNA120PhG_R_6_6, BNA024MFG_R_7_5, BNA007SFG_L_7_4, BNA150PCun_R_4_2, BNA036IFG_R_6_4, BNA061PrG_L_6_5, BNA125SPL_L_5_1, BNA172INS_R_6_5, BNA030IFG_R_6_1, BNA137IPL_L_6_2, BNA134SPL_R_5_5, BNA213Amyg_L_2_2, BNA240Tha_R_8_5, BNA118PhG_R_6_5, BNA241Tha_L_8_6, BNA214Amyg_R_2_2, BNA216Hipp_R_2_1, BNA076STG_R_6_4, BNA227Str_L_6_5, BNA201OcG_L_4_2, BNA220Str_R_6_1, BNA228Str_R_6_5, BNA123pSTS_L_2_2, BNA197Cun_L_5_5, BNA190Cun_R_5_1,
Elastic net	77	BNA136IPL_R_6_1, BNA084MTG_R_4_2, BNA064PrG_R_6_6, BNA096ITG_R_7_4, BNA052OrG_R_6_6, BNA162PoG_R_4_4, BNA183CG_L_7_5, BNA185CG_L_7_6, BNA039IFG_L_6_6, BNA156PoG_R_4_1, BNA078STG_R_6_5, BNA022MFG_R_7_4, BNA184CG_R_7_5, BNA020MFG_R_7_3, BNA054PrG_R_6_1, BNA154PCun_R_4_4, BNA008SFG_R_7_4, BNA166INS_R_6_2, BNA153PCun_L_4_4, BNA053PrG_L_6_1, BNA147PCun_L_4_1, BNA068PCL_R_2_2, BNA058PrG_R_6_3, BNA155PoG_L_4_1, BNA029IFG_L_6_1, BNA132SPL_R_5_4, BNA021MFG_L_7_4, BNA171INS_L_6_5, BNA174INS_R_6_6, BNA044OrG_R_6_2, BNA009SFG_L_7_5, BNA001SFG_L_7_1, BNA080STG_R_6_6, BNA025MFG_L_7_6, BNA027MFG_L_7_7, BNA088MTG_R_4_4, BNA028MFG_R_7_7, BNA048OrG_R_6_4, BNA045OrG_L_6_3, BNA014SFG_R_7_7, BNA003SFG_L_7_2, BNA159PoG_L_4_3, BNA226Str_R_6_4, BNA013SFG_L_7_7, BNA202OcG_R_4_2, BNA120PhG_R_6_6, BNA112PhG_R_6_2, BNA091ITG_L_7_2, BNA196Cun_R_5_4, BNA037IFG_L_6_5, BNA070STG_R_6_1, BNA098ITG_R_7_5, BNA151PCun_L_4_3, BNA090ITG_R_7_1, BNA104FuG_R_3_1, BNA010SFG_R_7_5, BNA188CG_R_7_7, BNA006SFG_R_7_3, BNA079STG_L_6_6, BNA082MTG_R_4_1, BNA186CG_R_7_6, BNA026MFG_R_7_6, BNA225Str_L_6_4, BNA060PrG_R_6_4, BNA005SFG_L_7_3, BNA179CG_L_7_3, BNA083MTG_L_4_2, BNA011SFG_L_7_6, BNA092ITG_R_7_2, BNA161PoG_L_4_4, BNA015MFG_L_7_1, BNA145IPL_L_6_6, BNA193Cun_L_5_3, BNA215Hipp_L_2_1, BNA163INS_L_6_1, BNA146IPL_R_6_6, BNA164INS_R_6_1,
Lars	61	BNA192Cun_R_5_2, BNA243Tha_L_8_7, BNA231Tha_L_8_1, BNA246Tha_R_8_8, BNA245Tha_L_8_8, BNA244Tha_R_8_7, BNA237Tha_L_8_4, BNA236Tha_R_8_3, BNA238Tha_R_8_4, BNA185CG_L_7_6, BNA039IFG_L_6_6, BNA156PoG_R_4_1,

		BNA078STG_R_6_5, BNA022MFG_R_7_4, BNA184CG_R_7_5, BNA020MFG_R_7_3, BNA182CG_R_7_4, BNA018MFG_R_7_2, BNA165INS_L_6_2, BNA176CG_R_7_1, BNA181CG_L_7_4, BNA100ITG_R_7_6, BNA016MFG_R_7_1, BNA130SPL_R_5_3, BNA168INS_R_6_3, BNA068PCL_R_2_2, BNA058PrG_R_6_3, BNA155PoG_L_4_1, BNA029IFG_L_6_1, BNA132SPL_R_5_4, BNA021MFG_L_7_4, BNA171INS_L_6_5, BNA174INS_R_6_6, BNA208sOcG_R_2_1, BNA142IPL_R_6_4, BNA144IPL_R_6_5, BNA086MTG_R_4_3, BNA107FuG_L_3_3, BNA196Cun_R_5_4, BNA037IFG_L_6_5, BNA070STG_R_6_1, BNA098ITG_R_7_5, BNA151PCun_L_4_3, BNA090ITG_R_7_1, BNA033IFG_L_6_3, BNA066PCL_R_2_1, BNA205OcG_L_4_4, BNA128SPL_R_5_2, BNA169INS_L_6_4, BNA099ITG_L_7_6, BNA057PrG_L_6_3, BNA035IFG_L_6_4, BNA229Str_L_6_6, BNA221Str_L_6_2, BNA059PrG_L_6_4, BNA187CG_L_7_7, BNA024MFG_R_7_5, BNA007SFG_L_7_4, BNA150PCun_R_4_2, BNA036IFG_R_6_4, BNA061PrG_L_6_5,
Lasso	67	BNA126SPL_R_5_1, BNA133SPL_L_5_5, BNA195Cun_L_5_4, BNA032IFG_R_6_2, BNA199OcG_L_4_1, BNA167INS_L_6_3, BNA077STG_L_6_5, BNA085MTG_L_4_3, BNA127SPL_L_5_2, BNA122pSTS_R_2_1, BNA101ITG_L_7_7, BNA073STG_L_6_3, BNA067PCL_L_2_2, BNA050OrG_R_6_5, BNA124pSTS_R_2_2, BNA097ITG_L_7_5, BNA140IPL_R_6_3, BNA093ITG_L_7_3, BNA119PhG_L_6_6, BNA208sOcG_R_2_1, BNA142IPL_R_6_4, BNA144IPL_R_6_5, BNA086MTG_R_4_3, BNA107FuG_L_3_3, BNA075STG_L_6_4, BNA148PCun_R_4_1, BNA173INS_L_6_6, BNA223Str_L_6_3, BNA209sOcG_L_2_2, BNA189Cun_L_5_1, BNA031IFG_L_6_2, BNA163INS_L_6_1, BNA146IPL_R_6_6, BNA164INS_R_6_1, BNA109PhG_L_6_1, BNA072STG_R_6_2, BNA212Amyg_R_2_1, BNA117PhG_L_6_5, BNA239Tha_L_8_5, BNA217Hipp_L_2_2, BNA242Tha_R_8_6, BNA115PhG_L_6_4, BNA194Cun_R_5_3, BNA192Cun_R_5_2, BNA243Tha_L_8_7, BNA231Tha_L_8_1, BNA246Tha_R_8_8, BNA036IFG_R_6_4, BNA061PrG_L_6_5, BNA125SPL_L_5_1, BNA172INS_R_6_5, BNA030IFG_R_6_1, BNA137IPL_L_6_2, BNA134SPL_R_5_5, BNA213Amyg_L_2_2, BNA240Tha_R_8_5, BNA118PhG_R_6_5, BNA241Tha_L_8_6, BNA214Amyg_R_2_2, BNA216Hipp_R_2_1, BNA211Amyg_L_2_1, BNA110PhG_R_6_1, BNA116PhG_R_6_4, BNA191Cun_L_5_2, BNA218Hipp_R_2_2, BNA071STG_L_6_2, BNA232Tha_R_8_1,
Kernel Ridge	82	BNA244Tha_R_8_7, BNA237Tha_L_8_4, BNA236Tha_R_8_3, BNA238Tha_R_8_4, BNA062PrG_R_6_5, BNA202OcG_R_4_2, BNA120PhG_R_6_6, BNA112PhG_R_6_2, BNA091ITG_L_7_2, BNA196Cun_R_5_4, BNA037IFG_L_6_5, BNA070STG_R_6_1, BNA098ITG_R_7_5, BNA151PCun_L_4_3, BNA090ITG_R_7_1, BNA104FuG_R_3_1, BNA177CG_L_7_2, BNA171INS_L_6_5,

		BNA174INS_R_6_6, BNA208sOcG_R_2_1, BNA142IPL_R_6_4, BNA144IPL_R_6_5, BNA086MTG_R_4_3, BNA107FuG_L_3_3, BNA075STG_L_6_4, BNA148PCun_R_4_1, BNA173INS_L_6_6, BNA223Str_L_6_3, BNA078STG_R_6_5, BNA022MFG_R_7_4, BNA184CG_R_7_5, BNA020MFG_R_7_3, BNA182CG_R_7_4, BNA018MFG_R_7_2, BNA165INS_L_6_2, BNA176CG_R_7_1, BNA181CG_L_7_4, BNA100ITG_R_7_6, BNA016MFG_R_7_1, BNA130SPL_R_5_3, BNA168INS_R_6_3, BNA081MTG_L_4_1, BNA041OrG_L_6_1, BNA160PoG_R_4_3, BNA048OrG_R_6_4, BNA045OrG_L_6_3, BNA014SFG_R_7_7, BNA003SFG_L_7_2, BNA159PoG_L_4_3, BNA226Str_R_6_4, BNA013SFG_L_7_7, BNA012SFG_R_7_6, BNA055PrG_L_6_2, BNA138IPL_R_6_2, BNA087MTG_L_4_4, BNA051OrG_L_6_6, BNA139IPL_L_6_3, BNA180CG_R_7_3, BNA017MFG_L_7_2, BNA175CG_L_7_1, BNA108FuG_R_3_3, BNA019MFG_L_7_3, BNA079STG_L_6_6, BNA082MTG_R_4_1, BNA186CG_R_7_6, BNA026MFG_R_7_6, BNA225Str_L_6_4, BNA060PrG_R_6_4, BNA005SFG_L_7_3, BNA179CG_L_7_3, BNA083MTG_L_4_2, BNA011SFG_L_7_6, BNA092ITG_R_7_2, BNA161PoG_L_4_4, BNA015MFG_L_7_1, BNA063PrG_L_6_6, BNA056PrG_R_6_2, BNA135IPL_L_6_1, BNA095ITG_L_7_4, BNA035IFG_L_6_4, BNA229Str_L_6_6, BNA221Str_L_6_2,
Multitask Lasso	66	BNA080STG_R_6_6, BNA025MFG_L_7_6, BNA027MFG_L_7_7, BNA088MTG_R_4_4, BNA028MFG_R_7_7, BNA048OrG_R_6_4, BNA045OrG_L_6_3, BNA014SFG_R_7_7, BNA003SFG_L_7_2, BNA159PoG_L_4_3, BNA226Str_R_6_4, BNA013SFG_L_7_7, BNA012SFG_R_7_6, BNA055PrG_L_6_2, BNA138IPL_R_6_2, BNA053PrG_L_6_1, BNA147PCun_L_4_1, BNA068PCL_R_2_2, BNA058PrG_R_6_3, BNA155PoG_L_4_1, BNA029IFG_L_6_1, BNA132SPL_R_5_4, BNA021MFG_L_7_4, BNA171INS_L_6_5, BNA174INS_R_6_6, BNA208sOcG_R_2_1, BNA050OrG_R_6_5, BNA124pSTS_R_2_2, BNA097ITG_L_7_5, BNA140IPL_R_6_3, BNA093ITG_L_7_3, BNA119PhG_L_6_6, BNA062PrG_R_6_5, BNA202OcG_R_4_2, BNA120PhG_R_6_6, BNA112PhG_R_6_2, BNA091ITG_L_7_2, BNA220Str_R_6_1, BNA228Str_R_6_5, BNA123pSTS_L_2_2, BNA197Cun_L_5_5, BNA190Cun_R_5_1, BNA113PhG_L_6_3, BNA114PhG_R_6_3, BNA204OcG_R_4_3, BNA207sOcG_L_2_1, BNA089ITG_L_7_1, BNA219Str_L_6_1, BNA024MFG_R_7_5, BNA007SFG_L_7_4, BNA150PCun_R_4_2, BNA036IFG_R_6_4, BNA061PrG_L_6_5, BNA125SPL_L_5_1, BNA172INS_R_6_5, BNA030IFG_R_6_1, BNA137IPL_L_6_2, BNA134SPL_R_5_5, BNA213Amyg_L_2_2, BNA240Tha_R_8_5, BNA118PhG_R_6_5, BNA241Tha_L_8_6, BNA214Amyg_R_2_2, BNA192Cun_R_5_2, BNA243Tha_L_8_7, BNA231Tha_L_8_1,
Decision Tree	71	BNA057PrG_L_6_3, BNA040IFG_R_6_6, BNA111PhG_L_6_2, BNA157PoG_L_4_2, BNA203OcG_L_4_3, BNA200OcG_R_4_1,

		BNA198Cun_R_5_5, BNA206OcG_R_4_4, BNA094ITG_R_7_3, BNA034IFG_R_6_3, BNA103FuG_L_3_1, BNA170INS_R_6_4, BNA152PCun_R_4_3, BNA105FuG_L_3_2, BNA067PCL_L_2_2, BNA050OrG_R_6_5, BNA124pSTS_R_2_2, BNA097ITG_L_7_5, BNA140IPL_R_6_3, BNA093ITG_L_7_3, BNA119PhG_L_6_6, BNA062PrG_R_6_5, BNA202OcG_R_4_2, BNA120PhG_R_6_6, BNA112PhG_R_6_2, BNA091ITG_L_7_2, BNA196Cun_R_5_4, BNA223Str_L_6_3, BNA209sOcG_L_2_2, BNA189Cun_L_5_1, BNA031IFG_L_6_2, BNA178CG_R_7_2, BNA224Str_R_6_3, BNA131SPL_L_5_4, BNA210sOcG_R_2_2, BNA121pSTS_L_2_1, BNA143IPL_L_6_5, BNA129SPL_L_5_3, BNA049OrG_L_6_5, BNA106FuG_R_3_2, BNA141IPL_L_6_4, BNA149PCun_L_4_2, BNA115PhG_L_6_4, BNA194Cun_R_5_3, BNA192Cun_R_5_2, BNA243Tha_L_8_7, BNA231Tha_L_8_1, BNA246Tha_R_8_8, BNA245Tha_L_8_8, BNA244Tha_R_8_7, BNA237Tha_L_8_4, BNA236Tha_R_8_3, BNA238Tha_R_8_4, BNA225Str_L_6_4, BNA060PrG_R_6_4, BNA005SFG_L_7_3, BNA179CG_L_7_3, BNA083MTG_L_4_2, BNA011SFG_L_7_6, BNA092ITG_R_7_2, BNA161PoG_L_4_4, BNA015MFG_L_7_1, BNA063PrG_L_6_6, BNA056PrG_R_6_2, BNA135IPL_L_6_1, BNA095ITG_L_7_4, BNA035IFG_L_6_4, BNA229Str_L_6_6, BNA221Str_L_6_2, BNA059PrG_L_6_4, BNA187CG_L_7_7,
Stochastic Gradient Descent	78	BNA012SFG_R_7_6, BNA055PrG_L_6_2, BNA138IPL_R_6_2, BNA087MTG_L_4_4, BNA051OrG_L_6_6, BNA139IPL_L_6_3, BNA180CG_R_7_3, BNA017MFG_L_7_2, BNA175CG_L_7_1, BNA108FuG_R_3_3, BNA019MFG_L_7_3, BNA047OrG_L_6_4, BNA023MFG_L_7_5, BNA002SFG_R_7_1, BNA042OrG_R_6_1, BNA004SFG_R_7_2, BNA074STG_R_6_3, BNA136IPL_R_6_1, BNA084MTG_R_4_2, BNA222Str_R_6_2, BNA038IFG_R_6_5, BNA102ITG_R_7_7, BNA054PrG_R_6_1, BNA154PCun_R_4_4, BNA008SFG_R_7_4, BNA166INS_R_6_2, BNA153PCun_L_4_4, BNA053PrG_L_6_1, BNA147PCun_L_4_1, BNA068PCL_R_2_2, BNA058PrG_R_6_3, BNA155PoG_L_4_1, BNA029IFG_L_6_1, BNA132SPL_R_5_4, BNA021MFG_L_7_4, BNA091ITG_L_7_2, BNA196Cun_R_5_4, BNA037IFG_L_6_5, BNA070STG_R_6_1, BNA098ITG_R_7_5, BNA151PCun_L_4_3, BNA090ITG_R_7_1, BNA104FuG_R_3_1, BNA177CG_L_7_2, BNA158PoG_R_4_2, BNA033IFG_L_6_3, BNA066PCL_R_2_1, BNA205OcG_L_4_4, BNA128SPL_R_5_2, BNA169INS_L_6_4, BNA099ITG_L_7_6, BNA095ITG_L_7_4, BNA035IFG_L_6_4, BNA229Str_L_6_6, BNA221Str_L_6_2, BNA059PrG_L_6_4, BNA187CG_L_7_7, BNA024MFG_R_7_5, BNA007SFG_L_7_4, BNA150PCun_R_4_2, BNA036IFG_R_6_4, BNA061PrG_L_6_5, BNA125SPL_L_5_1, BNA172INS_R_6_5, BNA030IFG_R_6_1, BNA137IPL_L_6_2, BNA134SPL_R_5_5, BNA213Amyg_L_2_2, BNA240Tha_R_8_5, BNA118PhG_R_6_5, BNA241Tha_L_8_6, BNA214Amyg_R_2_2,

		BNA216Hipp_R_2_1, BNA211Amyg_L_2_1, BNA113PhG_L_6_3, BNA114PhG_R_6_3, BNA204OcG_R_4_3, BNA207sOcG_L_2_1,

Table S6 The number of ROIs commonly selected by the FVS and Boruta algorithms (overlaps between Tables S7 and S8) and their atlas names for each classification models. FVS: forward variable selection algorithm. Entries are sorted in order of descending accuracy values (the best to worst from top to bottom)**.**

Model	Number of ROIs	Name of ROIs
Random Forest	36	BNA230Str_R_6_6 , BNA120PhG_R_6_6 , BNA090ITG_R_7_1 , BNA119PhG_L_6_6 , BNA105FuG_L_3_2 , BNA111PhG_L_6_2 , BNA213Amyg_L_2_2 , BNA206OcG_R_4_4 , BNA151PCun_L_4_3 , BNA094ITG_R_7_3 , BNA107FuG_L_3_3 , BNA243Tha_L_8_7 , BNA170INS_R_6_4 , BNA240Tha_R_8_5 , BNA241Tha_L_8_6 , BNA190Cun_R_5_1 , BNA122pSTS_R_2_1 , BNA060PrG_R_6_4 , BNA180CG_R_7_3 , BNA069STG_L_6_1 , BNA164INS_R_6_1 , BNA157PoG_L_4_2 , BNA220Str_R_6_1 , BNA200OcG_R_4_1 , BNA129SPL_L_5_3 , BNA166INS_R_6_2 , BNA058PrG_R_6_3 , BNA057PrG_L_6_3 , BNA125SPL_L_5_1 , BNA032IFG_R_6_2 , BNA031IFG_L_6_2 , BNA126SPL_R_5_1 , BNA065PCL_L_2_1 , BNA029IFG_L_6_1 , BNA030IFG_R_6_1 , BNA186CG_R_7_6 ,
Extreme Gradient Boosting	31	BNA084MTG_R_4_2, BNA089ITG_L_7_1, BNA078STG_R_6_5, BNA104FuG_R_3_1, BNA055PrG_L_6_2, BNA205OcG_L_4_4, BNA095ITG_L_7_4, BNA118PhG_R_6_5, BNA211Amyg_L_2_1, BNA047OrG_L_6_4, BNA242Tha_R_8_6, BNA088MTG_R_4_4, BNA182CG_R_7_4, BNA098ITG_R_7_5, BNA176CG_R_7_1, BNA092ITG_R_7_2, BNA023MFG_L_7_5, BNA109PhG_L_6_1, BNA235Tha_L_8_3, BNA144IPL_R_6_5, BNA050OrG_R_6_5, BNA024MFG_R_7_5, BNA189Cun_L_5_1, BNA072STG_R_6_2, BNA012SFG_R_7_6, BNA178CG_R_7_2, BNA071STG_L_6_2, BNA201OcG_L_4_2, BNA046OrG_R_6_3, BNA202OcG_R_4_2, BNA027MFG_L_7_7,
Logistic Regression with the Absolute Norm L1	39	BNA121pSTS_L_2_1, BNA130SPL_R_5_3, BNA210sOcG_R_2_2, BNA140IPL_R_6_3, BNA123pSTS_L_2_2, BNA063PrG_L_6_6, BNA209sOcG_L_2_2, BNA163INS_L_6_1, BNA068PCL_R_2_2, BNA005SFG_L_7_3, BNA134SPL_R_5_5, BNA017MFG_L_7_2, BNA006SFG_R_7_3, BNA135IPL_L_6_1, BNA066PCL_R_2_1, BNA161PoG_L_4_4, BNA022MFG_R_7_4, BNA131SPL_L_5_4, BNA149PCun_L_4_2, BNA137IPL_L_6_2, BNA155PoG_L_4_1, BNA140IPL_R_6_3, BNA123pSTS_L_2_2, BNA063PrG_L_6_6, BNA209sOcG_L_2_2, BNA163INS_L_6_1, BNA068PCL_R_2_2, BNA005SFG_L_7_3, BNA134SPL_R_5_5, BNA017MFG_L_7_2, BNA006SFG_R_7_3, BNA135IPL_L_6_1, BNA066PCL_R_2_1, BNA161PoG_L_4_4, BNA022MFG_R_7_4, BNA131SPL_L_5_4, BNA149PCun_L_4_2, BNA137IPL_L_6_2, BNA155PoG_L_4_1,
Gradient Boosting	27	BNA229Str_L_6_6, BNA223Str_L_6_3, BNA195Cun_L_5_4, BNA226Str_R_6_4, BNA083MTG_L_4_2, BNA123pSTS_L_2_2,

		BNA063PrG_L_6_6, BNA209sOcG_L_2_2, BNA163INS_L_6_1, BNA068PCL_R_2_2, BNA005SFG_L_7_3, BNA134SPL_R_5_5, BNA017MFG_L_7_2, BNA006SFG_R_7_3, BNA135IPL_L_6_1, BNA066PCL_R_2_1, BNA161PoG_L_4_4, BNA022MFG_R_7_4, BNA131SPL_L_5_4, BNA183CG_L_7_5, BNA045OrG_L_6_3, BNA020MFG_R_7_3, BNA100ITG_R_7_6, BNA018MFG_R_7_2, BNA039IFG_L_6_6, BNA177CG_L_7_2, BNA148PCun_R_4_1,
Extremely Randomize d Trees	34	BNA036IFG_R_6_4, BNA040IFG_R_6_6, BNA048OrG_R_6_4, BNA102ITG_R_7_7, BNA054PrG_R_6_1, BNA076STG_R_6_4, BNA192Cun_R_5_2, BNA183CG_L_7_5, BNA045OrG_L_6_3, BNA100ITG_R_7_6, BNA039IFG_L_6_6, BNA177CG_L_7_2, BNA097ITG_L_7_5, BNA184CG_R_7_5, BNA158PoG_R_4_2, BNA124pSTS_R_2_2, BNA099ITG_L_7_6, BNA159PoG_L_4_3, BNA194Cun_R_5_3, BNA156PoG_R_4_1, BNA041OrG_L_6_1, BNA070STG_R_6_1, BNA185CG_L_7_6, BNA067PCL_L_2_2, BNA121pSTS_L_2_1, BNA123pSTS_L_2_2, BNA063PrG_L_6_6, BNA163INS_L_6_1, BNA068PCL_R_2_2, BNA116PhG_R_6_4, BNA151PCun_L_4_3, BNA190Cun_R_5_1, BNA241Tha_L_8_6, BNA161PoG_L_4_4,
Decision Tree	21	BNA086MTG_R_4_3, BNA221Str_L_6_2, BNA106FuG_R_3_2, BNA037IFG_L_6_5, BNA222Str_R_6_2, BNA108FuG_R_3_3, BNA038IFG_R_6_5, BNA093ITG_L_7_3, BNA218Hipp_R_2_2, BNA224Str_R_6_3, BNA225Str_L_6_4, BNA084MTG_R_4_2, BNA089ITG_L_7_1, BNA104FuG_R_3_1, BNA095ITG_L_7_4, BNA091ITG_L_7_2, BNA103FuG_L_3_1, BNA087MTG_L_4_4, BNA094ITG_R_7_3, BNA234Tha_R_8_2, BNA097ITG_L_7_5,
NaVØve Bayes	13	BNA001SFG_L_7_1, BNA203OcG_L_4_3, BNA192Cun_R_5_2, BNA183CG_L_7_5, BNA177CG_L_7_2, BNA148PCun_R_4_1, BNA002SFG_R_7_1, BNA004SFG_R_7_2, BNA195Cun_L_5_4, BNA167INS_L_6_3, BNA168INS_R_6_3, BNA215Hipp_L_2_1, BNA196Cun_R_5_4,

Table S7 The number of ROIs solely selected by the <u>FVS algorithm</u> and their atlas names for each classification models. FVS: forward variable selection algorithm. Entries are sorted in order of descending accuracy values (the best to worst from top to bottom)**.**

Model	Number of ROIs	Name of ROIs
Random Forest	87	BNA230Str_R_6_6 , BNA120PhG_R_6_6 , BNA090ITG_R_7_1 , BNA119PhG_L_6_6 , BNA105FuG_L_3_2 , BNA111PhG_L_6_2 , BNA213Amyg_L_2_2 , BNA206OcG_R_4_4 , BNA151PCun_L_4_3 , BNA094ITG_R_7_3 , BNA107FuG_L_3_3 , BNA243Tha_L_8_7 , BNA170INS_R_6_4 , BNA240Tha_R_8_5 , BNA241Tha_L_8_6 , BNA190Cun_R_5_1 , BNA077STG_L_6_5 , BNA116PhG_R_6_4 , BNA035IFG_L_6_4 , BNA187CG_L_7_7 , BNA051OrG_L_6_6 , BNA049OrG_L_6_5 , BNA115PhG_L_6_4 , BNA096ITG_R_7_4 , BNA171INS_L_6_5 , BNA237Tha_L_8_4 , BNA244Tha_R_8_7 , BNA175CG_L_7_1 , BNA188CG_R_7_7 , BNA114PhG_R_6_3 , BNA141IPL_L_6_4 , BNA239Tha_L_8_5 , BNA101ITG_L_7_7 , BNA085MTG_L_4_3 , BNA165INS_L_6_2 , BNA198Cun_R_5_5 , BNA061PrG_L_6_5 , BNA212Amyg_R_2_1 , BNA074STG_R_6_3 , BNA059PrG_L_6_4 , BNA197Cun_L_5_5 , BNA052OrG_R_6_6 , BNA056PrG_R_6_2 , BNA008SFG_R_7_4 , BNA231Tha_L_8_1 , BNA011SFG_L_7_6 , BNA082MTG_R_4_1 , BNA013SFG_L_7_7 , BNA009SFG_L_7_5 , BNA227Str_L_6_5 , BNA154PCun_R_4_4 , BNA245Tha_L_8_8 , BNA169INS_L_6_4 , BNA246Tha_R_8_8 , BNA228Str_R_6_5 , BNA064PrG_R_6_6 , BNA204OcG_R_4_3 , BNA199OcG_L_4_1 , BNA191Cun_L_5_2 , BNA153PCun_L_4_4 , BNA193Cun_L_5_3 , BNA127SPL_L_5_2 , BNA053PrG_L_6_1 , BNA179CG_L_7_3 , BNA138IPL_R_6_2 , BNA207sOcG_L_2_1 , BNA186CG_R_7_6 , BNA122pSTS_R_2_1 , BNA060PrG_R_6_4 , BNA180CG_R_7_3 , BNA069STG_L_6_1 , BNA164INS_R_6_1 , BNA157PoG_L_4_2 , BNA220Str_R_6_1 , BNA200OcG_R_4_1 , BNA129SPL_L_5_3 , BNA166INS_R_6_2 , BNA058PrG_R_6_3 , BNA057PrG_L_6_3 , BNA125SPL_L_5_1 BNA162PoG_R_4_4 , BNA032IFG_R_6_2 , BNA031IFG_L_6_2 , BNA126SPL_R_5_1 , BNA065PCL_L_2_1 , BNA029IFG_L_6_1 , BNA030IFG_R_6_1 ,
Extreme Gradient Boosting	92	BNA079STG_L_6_6, BNA224Str_R_6_3, BNA225Str_L_6_4, BNA084MTG_R_4_2, BNA089ITG_L_7_1, BNA078STG_R_6_5, BNA104FuG_R_3_1, BNA055PrG_L_6_2, BNA205OcG_L_4_4, BNA095ITG_L_7_4, BNA118PhG_R_6_5, BNA206OcG_R_4_4, BNA171INS_L_6_5, BNA166INS_R_6_2, BNA243Tha_L_8_7, BNA057PrG_L_6_3, BNA096ITG_R_7_4, BNA231Tha_L_8_1, BNA029IFG_L_6_1, BNA058PrG_R_6_3, BNA120PhG_R_6_6, BNA049OrG_L_6_5, BNA107FuG_L_3_3, BNA105FuG_L_3_2, BNA240Tha_R_8_5, BNA193Cun_L_5_3, BNA085MTG_L_4_3, BNA111PhG_L_6_2, BNA165INS_L_6_2, BNA239Tha_L_8_5,

		BNA211Amyg_L_2_1, BNA047OrG_L_6_4, BNA242Tha_R_8_6, BNA088MTG_R_4_4, BNA182CG_R_7_4, BNA098ITG_R_7_5, BNA176CG_R_7_1, BNA092ITG_R_7_2, BNA023MFG_L_7_5, BNA109PhG_L_6_1, BNA235Tha_L_8_3, BNA144IPL_R_6_5, BNA050OrG_R_6_5, BNA024MFG_R_7_5, BNA189Cun_L_5_1, BNA072STG_R_6_2, BNA012SFG_R_7_6, BNA178CG_R_7_2, BNA071STG_L_6_2, BNA201OcG_L_4_2, BNA046OrG_R_6_3, BNA202OcG_R_4_2, BNA027MFG_L_7_7, BNA115PhG_L_6_4, BNA175CG_L_7_1, BNA207sOcG_L_2_1, BNA129SPL_L_5_3, BNA125SPL_L_5_1, BNA077STG_L_6_5, BNA101ITG_L_7_7, BNA212Amyg_R_2_1, BNA141IPL_L_6_4, BNA015MFG_L_7_1, BNA048OrG_R_6_4, BNA102ITG_R_7_7, BNA054PrG_R_6_1, BNA010SFG_R_7_5, BNA042OrG_R_6_1, BNA145IPL_L_6_6, BNA208sOcG_R_2_1, BNA028MFG_R_7_7, BNA128SPL_R_5_2, BNA234Tha_R_8_2, BNA097ITG_L_7_5, BNA184CG_R_7_5, BNA034IFG_R_6_3, BNA158PoG_R_4_2, BNA139IPL_L_6_3, BNA007SFG_L_7_4, BNA124pSTS_R_2_2, BNA099ITG_L_7_6, BNA159PoG_L_4_3, BNA003SFG_L_7_2, BNA146IPL_R_6_6, BNA194Cun_R_5_3, BNA219Str_L_6_1, BNA026MFG_R_7_6, BNA156PoG_R_4_1, BNA041OrG_L_6_1, BNA021MFG_L_7_4, BNA070STG_R_6_1, BNA185CG_L_7_6,
Logistic Regression with the Absolute Norm L1	98	BNA121pSTS_L_2_1, BNA130SPL_R_5_3, BNA210sOcG_R_2_2, BNA140IPL_R_6_3, BNA123pSTS_L_2_2, BNA063PrG_L_6_6, BNA209sOcG_L_2_2, BNA163INS_L_6_1, BNA068PCL_R_2_2, BNA005SFG_L_7_3, BNA134SPL_R_5_5, BNA017MFG_L_7_2, BNA006SFG_R_7_3, BNA135IPL_L_6_1, BNA066PCL_R_2_1, BNA161PoG_L_4_4, BNA022MFG_R_7_4, BNA131SPL_L_5_4, BNA149PCun_L_4_2, BNA137IPL_L_6_2, BNA155PoG_L_4_1, BNA160PoG_R_4_3, BNA150PCun_R_4_2, BNA132SPL_R_5_4, BNA233Tha_L_8_2, BNA098ITG_R_7_5, BNA176CG_R_7_1, BNA092ITG_R_7_2, BNA023MFG_L_7_5, BNA109PhG_L_6_1, BNA235Tha_L_8_3, BNA144IPL_R_6_5, BNA050OrG_R_6_5, BNA024MFG_R_7_5, BNA189Cun_L_5_1, BNA072STG_R_6_2, BNA012SFG_R_7_6, BNA178CG_R_7_2, BNA071STG_L_6_2, BNA201OcG_L_4_2, BNA046OrG_R_6_3, BNA202OcG_R_4_2, BNA027MFG_L_7_7, BNA043OrG_L_6_2, BNA236Tha_R_8_3, BNA033IFG_L_6_3, BNA172INS_R_6_5, BNA044OrG_R_6_2, BNA001SFG_L_7_1, BNA036IFG_R_6_4, BNA014SFG_R_7_7, BNA151PCun_L_4_3, BNA190Cun_R_5_1, BNA241Tha_L_8_6, BNA179CG_L_7_3, BNA213Amyg_L_2_2, BNA009SFG_L_7_5, BNA064PrG_R_6_6, BNA228Str_R_6_5, BNA059PrG_L_6_4, BNA154PCun_R_4_4, BNA246Tha_R_8_8, BNA169INS_L_6_4, BNA061PrG_L_6_5, BNA122pSTS_R_2_1, BNA069STG_L_6_1, BNA032IFG_R_6_2, BNA188CG_R_7_7, BNA227Str_L_6_5, BNA094ITG_R_7_3, BNA114PhG_R_6_3, BNA220Str_R_6_1, BNA060PrG_R_6_4, BNA082MTG_R_4_1, BNA138IPL_R_6_2,

		BNA074STG_R_6_3, BNA187CG_L_7_7, BNA164INS_R_6_1, BNA162PoG_R_4_4, BNA115PhG_L_6_4, BNA140IPL_R_6_3, BNA123pSTS_L_2_2, BNA063PrG_L_6_6, BNA209sOcG_L_2_2, BNA163INS_L_6_1, BNA068PCL_R_2_2, BNA005SFG_L_7_3, BNA134SPL_R_5_5, BNA017MFG_L_7_2, BNA006SFG_R_7_3, BNA135IPL_L_6_1, BNA066PCL_R_2_1, BNA161PoG_L_4_4, BNA022MFG_R_7_4, BNA131SPL_L_5_4, BNA149PCun_L_4_2, BNA137IPL_L_6_2, BNA155PoG_L_4_1,
Gradient Boosting	76	BNA229Str_L_6_6, BNA223Str_L_6_3, BNA195Cun_L_5_4, BNA226Str_R_6_4, BNA083MTG_L_4_2, BNA086MTG_R_4_3, BNA221Str_L_6_2, BNA167INS_L_6_3, BNA106FuG_R_3_2, BNA037IFG_L_6_5, BNA168INS_R_6_3, BNA215Hipp_L_2_1, BNA196Cun_R_5_4, BNA136IPL_R_6_1, BNA222Str_R_6_2, BNA108FuG_R_3_3, BNA038IFG_R_6_5, BNA112PhG_R_6_2, BNA093ITG_L_7_3, BNA218Hipp_R_2_2, BNA079STG_L_6_6, BNA224Str_R_6_3, BNA225Str_L_6_4, BNA084MTG_R_4_2, BNA089ITG_L_7_1, BNA078STG_R_6_5, BNA104FuG_R_3_1, BNA055PrG_L_6_2, BNA205OcG_L_4_4, BNA095ITG_L_7_4, BNA105FuG_L_3_2, BNA240Tha_R_8_5, BNA193Cun_L_5_3, BNA085MTG_L_4_3, BNA111PhG_L_6_2, BNA165INS_L_6_2, BNA239Tha_L_8_5, BNA035IFG_L_6_4, BNA119PhG_L_6_6, BNA011SFG_L_7_6, BNA053PrG_L_6_1, BNA245Tha_L_8_8, BNA056PrG_R_6_2, BNA031IFG_L_6_2, BNA199OcG_L_4_1, BNA052OrG_R_6_6, BNA126SPL_R_5_1, BNA127SPL_L_5_2, BNA030IFG_R_6_1, BNA170INS_R_6_4, BNA244Tha_R_8_7, BNA051OrG_L_6_6, BNA191Cun_L_5_2, BNA157PoG_L_4_2, BNA123pSTS_L_2_2, BNA063PrG_L_6_6, BNA209sOcG_L_2_2, BNA163INS_L_6_1, BNA068PCL_R_2_2, BNA005SFG_L_7_3, BNA134SPL_R_5_5, BNA017MFG_L_7_2, BNA006SFG_R_7_3, BNA135IPL_L_6_1, BNA066PCL_R_2_1, BNA161PoG_L_4_4, BNA022MFG_R_7_4, BNA131SPL_L_5_4, BNA183CG_L_7_5, BNA045OrG_L_6_3, BNA020MFG_R_7_3, BNA100ITG_R_7_6, BNA018MFG_R_7_2, BNA039IFG_L_6_6, BNA177CG_L_7_2, BNA148PCun_R_4_1,
Extremely Randomize d Trees	81	BNA036IFG_R_6_4, BNA014SFG_R_7_7, BNA040IFG_R_6_6, BNA015MFG_L_7_1, BNA048OrG_R_6_4, BNA102ITG_R_7_7, BNA054PrG_R_6_1, BNA010SFG_R_7_5, BNA232Tha_R_8_1, BNA203OcG_L_4_3, BNA238Tha_R_8_4, BNA133SPL_L_5_5, BNA076STG_R_6_4, BNA016MFG_R_7_1, BNA143IPL_L_6_5, BNA019MFG_L_7_3, BNA192Cun_R_5_2, BNA183CG_L_7_5, BNA045OrG_L_6_3, BNA020MFG_R_7_3, BNA100ITG_R_7_6, BNA018MFG_R_7_2, BNA039IFG_L_6_6, BNA177CG_L_7_2, BNA128SPL_R_5_2, BNA234Tha_R_8_2, BNA097ITG_L_7_5, BNA184CG_R_7_5, BNA034IFG_R_6_3, BNA158PoG_R_4_2, BNA139IPL_L_6_3, BNA007SFG_L_7_4, BNA124pSTS_R_2_2, BNA099ITG_L_7_6, BNA159PoG_L_4_3, BNA003SFG_L_7_2,

		BNA146IPL_R_6_6, BNA194Cun_R_5_3, BNA219Str_L_6_1, BNA026MFG_R_7_6, BNA156PoG_R_4_1, BNA041OrG_L_6_1, BNA021MFG_L_7_4, BNA070STG_R_6_1, BNA185CG_L_7_6, BNA067PCL_L_2_2, BNA121pSTS_L_2_1, BNA130SPL_R_5_3, BNA210sOcG_R_2_2, BNA140IPL_R_6_3, BNA123pSTS_L_2_2, BNA063PrG_L_6_6, BNA209sOcG_L_2_2, BNA163INS_L_6_1, BNA068PCL_R_2_2, BNA090ITG_R_7_1, BNA204OcG_R_4_3, BNA013SFG_L_7_7, BNA237Tha_L_8_4, BNA116PhG_R_6_4, BNA198Cun_R_5_5, BNA180CG_R_7_3, BNA200OcG_R_4_1, BNA008SFG_R_7_4, BNA186CG_R_7_6, BNA151PCun_L_4_3, BNA190Cun_R_5_1, BNA241Tha_L_8_6, BNA179CG_L_7_3, BNA213Amyg_L_2_2, BNA009SFG_L_7_5, BNA064PrG_R_6_6, BNA228Str_R_6_5, BNA059PrG_L_6_4, BNA154PCun_R_4_4, BNA246Tha_R_8_8, BNA169INS_L_6_4, BNA061PrG_L_6_5, BNA122pSTS_R_2_1, BNA161PoG_L_4_4, BNA022MFG_R_7_4,
Decision Tree	68	BNA086MTG_R_4_3, BNA221Str_L_6_2, BNA167INS_L_6_3, BNA106FuG_R_3_2, BNA037IFG_L_6_5, BNA168INS_R_6_3, BNA215Hipp_L_2_1, BNA196Cun_R_5_4, BNA136IPL_R_6_1, BNA222Str_R_6_2, BNA108FuG_R_3_3, BNA038IFG_R_6_5, BNA112PhG_R_6_2, BNA093ITG_L_7_3, BNA218Hipp_R_2_2, BNA079STG_L_6_6, BNA224Str_R_6_3, BNA225Str_L_6_4, BNA084MTG_R_4_2, BNA089ITG_L_7_1, BNA078STG_R_6_5, BNA104FuG_R_3_1, BNA055PrG_L_6_2, BNA205OcG_L_4_4, BNA095ITG_L_7_4, BNA118PhG_R_6_5, BNA174INS_R_6_6, BNA091ITG_L_7_2, BNA216Hipp_R_2_1, BNA214Amyg_R_2_2, BNA103FuG_L_3_1, BNA173INS_L_6_6, BNA080STG_R_6_6, BNA217Hipp_L_2_2, BNA075STG_L_6_4, BNA087MTG_L_4_4, BNA025MFG_L_7_6, BNA142IPL_R_6_4, BNA181CG_L_7_4, BNA113PhG_L_6_3, BNA062PrG_R_6_5, BNA117PhG_L_6_5, BNA081MTG_L_4_1, BNA094ITG_R_7_3, BNA114PhG_R_6_3, BNA220Str_R_6_1, BNA060PrG_R_6_4, BNA082MTG_R_4_1, BNA138IPL_R_6_2, BNA074STG_R_6_3, BNA187CG_L_7_7, BNA164INS_R_6_1, BNA162PoG_R_4_4, BNA115PhG_L_6_4, BNA175CG_L_7_1, BNA207sOcG_L_2_1, BNA129SPL_L_5_3, BNA125SPL_L_5_1, BNA077STG_L_6_5, BNA101ITG_L_7_7, BNA212Amyg_R_2_1, BNA141IPL_L_6_4, BNA128SPL_R_5_2, BNA234Tha_R_8_2, BNA097ITG_L_7_5, BNA184CG_R_7_5, BNA034IFG_R_6_3, BNA158PoG_R_4_2,
Nav√Øve Bayes	53	BNA044OrG_R_6_2, BNA001SFG_L_7_1, BNA036IFG_R_6_4, BNA014SFG_R_7_7, BNA040IFG_R_6_6, BNA015MFG_L_7_1, BNA048OrG_R_6_4, BNA102ITG_R_7_7, BNA054PrG_R_6_1, BNA010SFG_R_7_5, BNA232Tha_R_8_1, BNA203OcG_L_4_3, BNA238Tha_R_8_4, BNA133SPL_L_5_5, BNA076STG_R_6_4, BNA016MFG_R_7_1, BNA143IPL_L_6_5, BNA019MFG_L_7_3, BNA192Cun_R_5_2, BNA183CG_L_7_5, BNA045OrG_L_6_3, BNA020MFG_R_7_3, BNA100ITG_R_7_6, BNA018MFG_R_7_2,

		BNA039IFG_L_6_6, BNA177CG_L_7_2, BNA148PCun_R_4_1, BNA002SFG_R_7_1, BNA004SFG_R_7_2, BNA223Str_L_6_3, BNA195Cun_L_5_4, BNA226Str_R_6_4, BNA083MTG_L_4_2, BNA086MTG_R_4_3, BNA221Str_L_6_2, BNA167INS_L_6_3, BNA106FuG_R_3_2, BNA037IFG_L_6_5, BNA168INS_R_6_3, BNA215Hipp_L_2_1, BNA196Cun_R_5_4, BNA136IPL_R_6_1, BNA222Str_R_6_2, BNA108FuG_R_3_3, BNA038IFG_R_6_5, BNA112PhG_R_6_2, BNA093ITG_L_7_3, BNA218Hipp_R_2_2, BNA079STG_L_6_6, BNA224Str_R_6_3, BNA225Str_L_6_4, BNA084MTG_R_4_2, BNA089ITG_L_7_1,

Table S8 The number of ROIs solely selected by the Boruta algorithm and their atlas names for each classification models. Entries are sorted in order of descending accuracy values (the best to worst from top to bottom).

Model	Number of ROIs	Name of ROIs
Random Forest	73	BNA230Str_R_6_6, BNA120PhG_R_6_6, BNA090ITG_R_7_1, BNA119PhG_L_6_6, BNA105FuG_L_3_2, BNA111PhG_L_6_2, BNA213Amyg_L_2_2, BNA206OcG_R_4_4, BNA151PCun_L_4_3, BNA094ITG_R_7_3, BNA107FuG_L_3_3, BNA243Tha_L_8_7, BNA170INS_R_6_4, BNA240Tha_R_8_5, BNA241Tha_L_8_6, BNA190Cun_R_5_1, BNA116PhG_R_6_4, BNA035IFG_L_6_4, BNA167INS_L_6_3, BNA168INS_R_6_3, BNA172INS_R_6_5, BNA173INS_L_6_6, BNA174INS_R_6_6, BNA176CG_R_7_1, BNA177CG_L_7_2, BNA178CG_R_7_2, BNA181CG_L_7_4, BNA182CG_R_7_4, BNA194Cun_R_5_3, BNA195Cun_L_5_4, BNA196Cun_R_5_4, BNA201OcG_L_4_2, BNA202OcG_R_4_2, BNA203OcG_L_4_3, BNA205OcG_L_4_4, BNA208sOcG_R_2_1, BNA209sOcG_L_2_2, BNA210sOcG_R_2_2, BNA211Amyg_L_2_1, BNA214Amyg_R_2_2, BNA215Hipp_L_2_1, BNA216Hipp_R_2_1, BNA217Hipp_L_2_2, BNA218Hipp_R_2_2, BNA219Str_L_6_1, BNA221Str_L_6_2, BNA222Str_R_6_2, BNA223Str_L_6_3, BNA224Str_R_6_3, BNA001SFG_L_7_1, BNA002SFG_R_7_1, BNA003SFG_L_7_2, BNA004SFG_R_7_2, BNA005SFG_L_7_3, BNA006SFG_R_7_3, BNA007SFG_L_7_4, BNA010SFG_R_7_5, BNA122pSTS_R_2_1, BNA060PrG_R_6_4, BNA180CG_R_7_3, BNA069STG_L_6_1, BNA157PoG_L_4_2, BNA220Str_R_6_1, BNA200OcG_R_4_1, BNA166INS_R_6_2, BNA058PrG_R_6_3, BNA057PrG_L_6_3, BNA032IFG_R_6_2, BNA031IFG_L_6_2, BNA126SPL_R_5_1, BNA065PCL_L_2_1, BNA029IFG_L_6_1, BNA030IFG_R_6_1,
Extreme Gradient Boosting	110	BNA084MTG_R_4_2, BNA089ITG_L_7_1, BNA078STG_R_6_5, BNA104FuG_R_3_1, BNA055PrG_L_6_2, BNA205OcG_L_4_4, BNA095ITG_L_7_4, BNA118PhG_R_6_5, BNA174INS_R_6_6, BNA091ITG_L_7_2, BNA216Hipp_R_2_1, BNA214Amyg_R_2_2, BNA103FuG_L_3_1, BNA173INS_L_6_6, BNA080STG_R_6_6, BNA217Hipp_L_2_2, BNA075STG_L_6_4, BNA087MTG_L_4_4, BNA025MFG_L_7_6, BNA142IPL_R_6_4, BNA181CG_L_7_4, BNA113PhG_L_6_3, BNA062PrG_R_6_5, BNA117PhG_L_6_5, BNA081MTG_L_4_1, BNA110PhG_R_6_1, BNA152PCun_R_4_3, BNA211Amyg_L_2_1, BNA047OrG_L_6_4, BNA242Tha_R_8_6, BNA088MTG_R_4_4, BNA182CG_R_7_4, BNA098ITG_R_7_5, BNA176CG_R_7_1, BNA092ITG_R_7_2, BNA023MFG_L_7_5, BNA109PhG_L_6_1, BNA235Tha_L_8_3, BNA144IPL_R_6_5, BNA050OrG_R_6_5, BNA024MFG_R_7_5, BNA189Cun_L_5_1,

		BNA072STG_R_6_2, BNA012SFG_R_7_6, BNA178CG_R_7_2, BNA071STG_L_6_2, BNA201OcG_L_4_2, BNA046OrG_R_6_3, BNA202OcG_R_4_2, BNA027MFG_L_7_7, BNA043OrG_L_6_2, BNA236Tha_R_8_3, BNA158PoG_R_4_2, BNA139IPL_L_6_3, BNA007SFG_L_7_4, BNA124pSTS_R_2_2, BNA099ITG_L_7_6, BNA159PoG_L_4_3, BNA003SFG_L_7_2, BNA146IPL_R_6_6, BNA194Cun_R_5_3, BNA219Str_L_6_1, BNA026MFG_R_7_6, BNA156PoG_R_4_1, BNA041OrG_L_6_1, BNA021MFG_L_7_4, BNA070STG_R_6_1, BNA185CG_L_7_6, BNA067PCL_L_2_2, BNA121pSTS_L_2_1, BNA130SPL_R_5_3, BNA210sOcG_R_2_2, BNA140IPL_R_6_3, BNA123pSTS_L_2_2, BNA063PrG_L_6_6, BNA209sOcG_L_2_2, BNA163INS_L_6_1, BNA068PCL_R_2_2, BNA005SFG_L_7_3, BNA134SPL_R_5_5, BNA017MFG_L_7_2, BNA006SFG_R_7_3, BNA135IPL_L_6_1, BNA066PCL_R_2_1, BNA161PoG_L_4_4, BNA022MFG_R_7_4, BNA131SPL_L_5_4, BNA149PCun_L_4_2, BNA137IPL_L_6_2, BNA155PoG_L_4_1, BNA160PoG_R_4_3, BNA045OrG_L_6_3, BNA020MFG_R_7_3, BNA100ITG_R_7_6, BNA018MFG_R_7_2, BNA039IFG_L_6_6, BNA177CG_L_7_2, BNA148PCun_R_4_1, BNA002SFG_R_7_1, BNA004SFG_R_7_2, BNA073STG_L_6_3, BNA147PCun_L_4_1, BNA042OrG_R_6_1, BNA145IPL_L_6_6, BNA208sOcG_R_2_1, BNA028MFG_R_7_7, BNA128SPL_R_5_2, BNA229Str_L_6_6, BNA223Str_L_6_3, BNA195Cun_L_5_4,
Logistic Regression with the Absolute Norm L1	88	BNA226Str_R_6_4, BNA083MTG_L_4_2, BNA086MTG_R_4_3, BNA221Str_L_6_2, BNA167INS_L_6_3, BNA106FuG_R_3_2, BNA037IFG_L_6_5, BNA168INS_R_6_3, BNA215Hipp_L_2_1, BNA196Cun_R_5_4, BNA136IPL_R_6_1, BNA222Str_R_6_2, BNA108FuG_R_3_3, BNA038IFG_R_6_5, BNA112PhG_R_6_2, BNA093ITG_L_7_3, BNA218Hipp_R_2_2, BNA079STG_L_6_6, BNA224Str_R_6_3, BNA225Str_L_6_4, BNA084MTG_R_4_2, BNA089ITG_L_7_1, BNA078STG_R_6_5, BNA104FuG_R_3_1, BNA055PrG_L_6_2, BNA205OcG_L_4_4, BNA095ITG_L_7_4, BNA118PhG_R_6_5, BNA174INS_R_6_6, BNA091ITG_L_7_2, BNA216Hipp_R_2_1, BNA214Amyg_R_2_2, BNA103FuG_L_3_1, BNA173INS_L_6_6, BNA080STG_R_6_6, BNA217Hipp_L_2_2, BNA172INS_R_6_5, BNA044OrG_R_6_2, BNA001SFG_L_7_1, BNA036IFG_R_6_4, BNA014SFG_R_7_7, BNA040IFG_R_6_6, BNA015MFG_L_7_1, BNA048OrG_R_6_4, BNA102ITG_R_7_7, BNA054PrG_R_6_1, BNA010SFG_R_7_5, BNA232Tha_R_8_1, BNA203OcG_L_4_3, BNA238Tha_R_8_4, BNA133SPL_L_5_5, BNA076STG_R_6_4, BNA016MFG_R_7_1, BNA143IPL_L_6_5, BNA019MFG_L_7_3, BNA192Cun_R_5_2, BNA183CG_L_7_5, BNA045OrG_L_6_3, BNA020MFG_R_7_3, BNA100ITG_R_7_6, BNA018MFG_R_7_2, BNA039IFG_L_6_6, BNA177CG_L_7_2, BNA148PCun_R_4_1, BNA002SFG_R_7_1, BNA004SFG_R_7_2, BNA073STG_L_6_3, BNA147PCun_L_4_1, BNA042OrG_R_6_1,

		BNA145IPL_L_6_6, BNA208sOcG_R_2_1, BNA028MFG_R_7_7, BNA128SPL_R_5_2, BNA234Tha_R_8_2, BNA097ITG_L_7_5, BNA184CG_R_7_5, BNA026MFG_R_7_6, BNA156PoG_R_4_1, BNA041OrG_L_6_1, BNA021MFG_L_7_4, BNA070STG_R_6_1, BNA185CG_L_7_6, BNA067PCL_L_2_2, BNA121pSTS_L_2_1, BNA130SPL_R_5_3, BNA210sOcG_R_2_2, BNA140IPL_R_6_3, BNA123pSTS_L_2_2,
Gradient Boosting	81	BNA158PoG_R_4_2, BNA139IPL_L_6_3, BNA007SFG_L_7_4, BNA124pSTS_R_2_2, BNA099ITG_L_7_6, BNA159PoG_L_4_3, BNA003SFG_L_7_2, BNA146IPL_R_6_6, BNA194Cun_R_5_3, BNA219Str_L_6_1, BNA026MFG_R_7_6, BNA156PoG_R_4_1, BNA041OrG_L_6_1, BNA021MFG_L_7_4, BNA070STG_R_6_1, BNA185CG_L_7_6, BNA067PCL_L_2_2, BNA121pSTS_L_2_1, BNA130SPL_R_5_3, BNA210sOcG_R_2_2, BNA140IPL_R_6_3, BNA123pSTS_L_2_2, BNA063PrG_L_6_6, BNA209sOcG_L_2_2, BNA163INS_L_6_1, BNA068PCL_R_2_2, BNA005SFG_L_7_3, BNA134SPL_R_5_5, BNA017MFG_L_7_2, BNA006SFG_R_7_3, BNA135IPL_L_6_1, BNA066PCL_R_2_1, BNA161PoG_L_4_4, BNA022MFG_R_7_4, BNA131SPL_L_5_4, BNA149PCun_L_4_2, BNA137IPL_L_6_2, BNA155PoG_L_4_1, BNA160PoG_R_4_3, BNA150PCun_R_4_2, BNA132SPL_R_5_4, BNA233Tha_L_8_2, BNA015MFG_L_7_1, BNA048OrG_R_6_4, BNA102ITG_R_7_7, BNA054PrG_R_6_1, BNA010SFG_R_7_5, BNA232Tha_R_8_1, BNA203OcG_L_4_3, BNA238Tha_R_8_4, BNA133SPL_L_5_5, BNA076STG_R_6_4, BNA016MFG_R_7_1, BNA143IPL_L_6_5, BNA019MFG_L_7_3, BNA192Cun_R_5_2, BNA183CG_L_7_5, BNA045OrG_L_6_3, BNA020MFG_R_7_3, BNA100ITG_R_7_6, BNA018MFG_R_7_2, BNA039IFG_L_6_6, BNA177CG_L_7_2, BNA148PCun_R_4_1, BNA002SFG_R_7_1, BNA004SFG_R_7_2, BNA073STG_L_6_3, BNA147PCun_L_4_1, BNA042OrG_R_6_1, BNA145IPL_L_6_6, BNA208sOcG_R_2_1, BNA028MFG_R_7_7, BNA128SPL_R_5_2, BNA234Tha_R_8_2, BNA097ITG_L_7_5, BNA184CG_R_7_5, BNA229Str_L_6_6, BNA223Str_L_6_3, BNA195Cun_L_5_4, BNA226Str_R_6_4, BNA083MTG_L_4_2,
Extremely Randomized Trees	102	BNA156PoG_R_4_1, BNA183CG_L_7_5, BNA185CG_L_7_6, BNA163INS_L_6_1, BNA182CG_R_7_4, BNA155PoG_L_4_1, BNA048OrG_R_6_4, BNA091ITG_L_7_2, BNA160PoG_R_4_3, BNA036IFG_R_6_4, BNA043OrG_L_6_2, BNA159PoG_L_4_3, BNA093ITG_L_7_3, BNA089ITG_L_7_1, BNA178CG_R_7_2, BNA168INS_R_6_3, BNA194Cun_R_5_3, BNA177CG_L_7_2, BNA092ITG_R_7_2, BNA095ITG_L_7_4, BNA047OrG_L_6_4, BNA073STG_L_6_3, BNA189Cun_L_5_1, BNA062PrG_R_6_5, BNA177CG_L_7_2, BNA243Tha_L_8_7, BNA181CG_L_7_4, BNA037IFG_L_6_5, BNA167INS_L_6_3, BNA192Cun_R_5_2, BNA094ITG_R_7_3, BNA182CG_R_7_4, BNA066PCL_R_2_1, BNA176CG_R_7_1, BNA075STG_L_6_4, BNA178CG_R_7_2,

		BNA099ITG_L_7_6, BNA181CG_L_7_4, BNA071STG_L_6_2, BNA044OrG_R_6_2, BNA170INS_R_6_4, BNA046OrG_R_6_3, BNA079STG_L_6_6, BNA045OrG_L_6_3, BNA173INS_L_6_6, BNA042OrG_R_6_1, BNA102ITG_R_7_7, BNA158PoG_R_4_2, BNA038IFG_R_6_5, BNA086MTG_R_4_3, BNA240Tha_R_8_5, BNA083MTG_L_4_2, BNA041OrG_L_6_1, BNA072STG_R_6_2, BNA040IFG_R_6_6, BNA067PCL_L_2_2, BNA078STG_R_6_5, BNA076STG_R_6_4, BNA063PrG_L_6_6, BNA080STG_R_6_6, BNA097ITG_L_7_5, BNA039IFG_L_6_6, BNA098ITG_R_7_5, BNA054PrG_R_6_1, BNA084MTG_R_4_2, BNA174INS_R_6_6, BNA167INS_L_6_3, BNA050OrG_R_6_5, BNA123pSTS_L_2_2, BNA107FuG_L_3_3, BNA117PhG_L_6_5, BNA081MTG_L_4_1, BNA184CG_R_7_5, BNA168INS_R_6_3, BNA108FuG_R_3_3, BNA174INS_R_6_6, BNA088MTG_R_4_4, BNA173INS_L_6_6, BNA172INS_R_6_5, BNA055PrG_L_6_2, BNA113PhG_L_6_3, BNA100ITG_R_7_6, BNA106FuG_R_3_2, BNA241Tha_L_8_6, BNA121pSTS_L_2_1, BNA176CG_R_7_1, BNA151PCun_L_4_3, BNA087MTG_L_4_4, BNA190Cun_R_5_1, BNA103FuG_L_3_1, BNA118PhG_R_6_5, BNA110PhG_R_6_1, BNA104FuG_R_3_1, BNA124pSTS_R_2_2, BNA172INS_R_6_5, BNA070STG_R_6_1, BNA035IFG_L_6_4, BNA161PoG_L_4_4, BNA068PCL_R_2_2, BNA116PhG_R_6_4, BNA112PhG_R_6_2, BNA109PhG_L_6_1,
Decision Tree	77	BNA004SFG_R_7_2, BNA036IFG_R_6_4, BNA038IFG_R_6_5, BNA012SFG_R_7_6, BNA023MFG_L_7_5, BNA003SFG_L_7_2, BNA098ITG_R_7_5, BNA007SFG_L_7_4, BNA006SFG_R_7_3, BNA100ITG_R_7_6, BNA097ITG_L_7_5, BNA235Tha_L_8_3, BNA015MFG_L_7_1, BNA002SFG_R_7_1, BNA234Tha_R_8_2, BNA099ITG_L_7_6, BNA102ITG_R_7_7, BNA039IFG_L_6_6, BNA020MFG_R_7_3, BNA041OrG_L_6_1, BNA190Cun_R_5_1, BNA236Tha_R_8_3, BNA014SFG_R_7_7, BNA040IFG_R_6_6, BNA240Tha_R_8_5, BNA001SFG_L_7_1, BNA019MFG_L_7_3, BNA021MFG_L_7_4, BNA106FuG_R_3_2, BNA022MFG_R_7_4, BNA116PhG_R_6_4, BNA086MTG_R_4_3, BNA017MFG_L_7_2, BNA109PhG_L_6_1, BNA005SFG_L_7_3, BNA092ITG_R_7_2, BNA218Hipp_R_2_2, BNA089ITG_L_7_1, BNA084MTG_R_4_2, BNA083MTG_L_4_2, BNA087MTG_L_4_4, BNA103FuG_L_3_1, BNA104FuG_R_3_1, BNA091ITG_L_7_2, BNA018MFG_R_7_2, BNA224Str_R_6_3, BNA107FuG_L_3_3, BNA241Tha_L_8_6, BNA206OcG_R_4_4, BNA088MTG_R_4_4, BNA037IFG_L_6_5, BNA225Str_L_6_4, BNA090ITG_R_7_1, BNA232Tha_R_8_1, BNA095ITG_L_7_4, BNA229Str_L_6_6, BNA016MFG_R_7_1, BNA213Amyg_L_2_2, BNA108FuG_R_3_3, BNA120PhG_R_6_6, BNA219Str_L_6_1, BNA094ITG_R_7_3, BNA233Tha_L_8_2, BNA170INS_R_6_4, BNA093ITG_L_7_3, BNA221Str_L_6_2, BNA110PhG_R_6_1, BNA151PCun_L_4_3, BNA105FuG_L_3_2, BNA230Str_R_6_6, BNA243Tha_L_8_7, BNA226Str_R_6_4,

		BNA223Str_L_6_3, BNA010SFG_R_7_5, BNA222Str_R_6_2, BNA111PhG_L_6_2, BNA119PhG_L_6_6,
Nav√Øve Bayes	64	BNA181CG_L_7_4, BNA183CG_L_7_5, BNA211Amyg_L_2_1, BNA203OcG_L_4_3, BNA214Amyg_R_2_2, BNA205OcG_L_4_4, BNA217Hipp_L_2_2, BNA195Cun_L_5_4, BNA176CG_R_7_1, BNA182CG_R_7_4, BNA189Cun_L_5_1, BNA174INS_R_6_6, BNA210sOcG_R_2_2, BNA152PCun_R_4_3, BNA196Cun_R_5_4, BNA003SFG_L_7_2, BNA192Cun_R_5_2, BNA209sOcG_L_2_2, BNA177CG_L_7_2, BNA172INS_R_6_5, BNA184CG_R_7_5, BNA194Cun_R_5_3, BNA001SFG_L_7_1, BNA155PoG_L_4_1, BNA002SFG_R_7_1, BNA178CG_R_7_2, BNA216Hipp_R_2_1, BNA147PCun_L_4_1, BNA185CG_L_7_6, BNA202OcG_R_4_2, BNA194Cun_R_5_3, BNA215Hipp_L_2_1, BNA149PCun_L_4_2, BNA167INS_L_6_3, BNA163INS_L_6_1, BNA146IPL_R_6_6, BNA196Cun_R_5_4, BNA214Amyg_R_2_2, BNA215Hipp_L_2_1, BNA202OcG_R_4_2, BNA209sOcG_L_2_2, BNA148PCun_R_4_1, BNA208sOcG_R_2_1, BNA150PCun_R_4_2, BNA173INS_L_6_6, BNA145IPL_L_6_6, BNA205OcG_L_4_4, BNA208sOcG_R_2_1, BNA168INS_R_6_3, BNA211Amyg_L_2_1, BNA201OcG_L_4_2, BNA007SFG_L_7_4, BNA201OcG_L_4_2, BNA006SFG_R_7_3, BNA203OcG_L_4_3, BNA195Cun_L_5_4, BNA210sOcG_R_2_2, BNA161PoG_L_4_4, BNA159PoG_L_4_3, BNA004SFG_R_7_2, BNA005SFG_L_7_3, BNA156PoG_R_4_1, BNA160PoG_R_4_3, BNA158PoG_R_4_2,
